# Thymopentin alleviates premature ovarian failure in mice by activating YY2/Lin28A and inhibiting the expression of let‐7 family microRNAs

**DOI:** 10.1111/cpr.13089

**Published:** 2021-06-28

**Authors:** Te Liu, Fangyuan Jing, Peirong Huang, Zixiang Geng, Jianghong Xu, Jiahui Li, Danping Chen, Ying Zhu, Zhenxin Wang, Willian Huang, Chuan Chen

**Affiliations:** ^1^ Shanghai Geriatric Institute of Chinese Medicine Shanghai University of Traditional Chinese Medicine Shanghai China; ^2^ Department of Ophthalmology Shanghai General Hospital Shanghai Jiao Tong University School of Medicine Shanghai China; ^3^ Center for Advanced Vision Science University of Virginia School of Medicine Charlottesville VA USA; ^4^ Department of Acupuncture Shanghai General Hospital Shanghai Jiao Tong University School of Medicine Shanghai China; ^5^ Department of gynaecology Jingan Hospital of Traditional Chinese Medicine Shanghai China; ^6^ Department of Laboratory Medicine of Zhongshan Hospital and institute of Biomedical Science Fudan University Shanghai China; ^7^ Hainan Zhonghe Pharmaceutical Co., Ltd Haikou China

**Keywords:** exosome, high‐fat and high‐sugar (HFHS), poly(lactic‐*co*‐glycolic acid [PLGA]) nanoparticle, premature ovarian failure, Yin Yang 2 (YY2)

## Abstract

**Objective:**

Thymopentin (5TP) significantly improved typical murine premature ovarian failure (POF) symptoms induced by a high‐fat and high‐sugar (HFHS) diet. However, its effect and mechanism remain unclear.

**Materials and methods:**

RNA‐Seq was used to detect the differentially expressed genes among each group. HFHS‐induced POF mouse model was generated and injected with siRNA using Poly (lactic‐co‐glycolic acid) (PLGA) as a carrier.

**Results:**

RNA‐Seq suggested that 5TP promoted the expression of Yin Yang 2 (YY2) in mouse ovarian granulosa cell (mOGC) of HFHS‐POF mice. Luciferase reporter assay indicated that 5TP promoted the binding of YY2 to the specific sequence C(C/T)AT(G/C)(G/T) on the Lin28A promoter and promoted Lin28A transcription and expression. We continuously injected PLGA‐cross‐linked siRNA nanoparticles targeting YY2 into HFHS‐POF mice (siYY2@PLGA), which significantly reduced the therapeutic effect of 5TP. siYY2@PLGA injection also significantly attenuated the upregulation of Lin28a expression in mOGCs induced by 5TP and enhanced the expression of let‐7 family microRNAs, thereby inhibiting the proliferation and division of mOGCs. qPCR results showed that there was a significant difference in the expression levels of exosome‐derived Yy2 mRNAs between POF patients and normal women, and that there was a specific correlation between the expression level of exosome‐derived Yy2 and the peripheral concentrations of the blood hormones pregnenolone, progesterone and oestradiol.

**Conclusions:**

Thymopentin promotes the transcriptional activation of Lin28A via stimulating transcription factor YY2 expression, inhibits the activity of let‐7 family microRNAs and alleviates the ageing of ovarian granulosa cells, ultimately achieving a therapeutic effect on POF in mice.

## Graphical abstract

Thymopentin promotes the transcriptional activation of Lin28A via stimulating transcription factor YY2 expression, inhibit the activity of let‐7 family microRNAs, and alleviate the aging of ovarian granulosa cells, ultimately achieving a therapeutic effect on POF in mice.

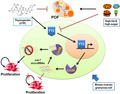

## 1 INTRODUCTION

Premature ovarian failure (POF) is a disorder characterized by amenorrhea, infertility, low oestrogen levels, high concentrations of gonadotropins and a lack of mature ovarian follicles before the age of 40, and it represents one of the most common causes of female infertility.^1^, ^2^, ^3^, ^4^ The occurrence of POF is closely related to the state and quality of ovarian granulosa cells (OGCs),^1^, ^5^, ^6^ and ageing and apoptosis of OGCs are some of the essential reasons for the decline in ovarian reserve function.^5^, ^6^ Many investigators have found that high‐fat and high‐sugar (HFHS) diets not only increase the risk of obesity,^7^, ^8^, ^9^ tumours and cardiovascular disease but also seriously affect ovarian function and oocyte quality.^10^, ^11^, ^12^ However, the mechanisms underlying OGC ageing and POF as caused by obesity and HFHS diet still require further study. We previously observed that oxidative stress damage of OGCs is one of the primary causes for their deterioration.^1^ We also reported epigenetic mechanisms of action subserving the dietary effects of HFHS, leading to ovarian granulosa cell ageing and premature ovarian failure by inhibiting endogenous miR‐146, activating the DAB2IP/ASK1/p38‐signalling pathway and inducing γ‐H2A.X phosphorylation modification.^13^ All of these actions show that HFHS can elevate the risk for POF.

Aspartic acid thymopentin (TP‐5, 5TP) is composed of arginine, lysine, aspartic acid, valine and tyrosine,^14^, ^15^, ^16^, ^17^, ^18^ and its chemical name is N‐[N‐[N‐[NL‐arginyl‐L‐lysyl]‐L‐α‐aspartyl]‐L‐valinyl]‐L‐tyrosine.^14^, ^15^, ^16^, ^17^, ^18^ The molecular formula is C_30_H_49_N_9_O_9_, and the molecular weight is 679.77. TP‐5 is a valuable component of thymopoietin II in thymic secretions.^14^, ^15^, ^16^, ^17^, ^18^ Thymopoietin II is a single polypeptide isolated from thymic hormone and is composed of 49 amino acids; however, the peptide chain composed of 5 amino acids displays the same physiologic function as thymopoietin II, and thus, this pentapeptide is called thymopentin.^14^, ^15^, ^16^, ^17^, ^18^ Some studies have shown that TP‐5 exerts a significant immunomodulatory effect. For example, it can increase the levels of cAMP, promote T‐cell differentiation, and bind to T‐cell‐specific receptors to elevate the levels of intracellular GMP—thus inducing a series of intracellular reactions and playing a role in regulating the immune function of the body.^14^, ^15^, ^16^, ^17^, ^18^ TP‐5 can additionally induce T‐cell differentiation, promote the development, maturation and activation of T lymphocyte subsets and restore the proportion of CD4/CD8 to normal.^14^, ^15^, ^16^, ^17^, ^18^ TP‐5 has a significant effect on promoting the reconstruction of immune function in malignant tumour patients after radiotherapy and chemotherapy, and improves the immune system of elderly patients with low immune function.^14^, ^15^, ^16^, ^17^, ^18^ TP‐5 also manifests a specific therapeutic effect on autoimmune diseases—including rheumatoid arthritis—and we uncovered no extant reports on treatments for POF using TP‐5.

Based on the current data and the HFHS‐induced POF mouse model, we herein aimed to explore the protective effects of TP‐5 on ovarian granulosa cell ageing and injury, as well as ascertain the underlying molecular biologic mechanism(s), and aspired to evaluate the therapeutic effects of TP‐5 on POF.

## 2 MATERIALS AND METHODS

A detailed description of all materials and methods can be found in [Link], [Link], [Link].

### 2.1 HFHS‐POF model and TP‐5 treatment

This model derives from prbeviously published methods.^7^, ^13^, ^19^, ^20^ Briefly, 10‐week‐old female C57BL/6 mice (n = 30) were purchased from the Experimental Animal Centre of the Shanghai University of Traditional Chinese Medicine. Mice were randomly allocated to three groups, with ten mice per group. WT mice in the blank control group were fed with a normal diet without any intervention, while the mice in the treatment group (POF‐5TP) were fed with a high‐fat diet (8 g/kg) and received 200 μL of 30% fructose and an intraperitoneal injection of 5 mg/kg TP‐5 once per day (Hainan Zhonghe Pharmaceutical Co., Ltd). The mice in the model group (POF‐Saline) were fed with a high‐fat diet (8 g/kg) and received 200 μL of 30% fructose and an intraperitoneal injection of normal saline once per day. Mice in each group were administered treatment continuously for 30 days. Our protocol was approved by the Ethics Committee of the Shanghai Institute of Traditional Chinese Medicine and Geriatrics (no. SHAGESYDW202009). All of the experiments complied with the regulations on Experimental Animals of the State Science and Technology Commission of China.

### 2.2 SiRNA loading of PLGA nanoparticles and interventions in animal models

Our methods were performed according to previous reports.^13^, ^21^, ^22^, ^23^ Briefly, YY2‐siRNA (siYY2) and random control (siMock) oligoRNAs were synthesized by GenePharma (GenePharma). The PLGA (MedChemExpress) was dissolved overnight in methylene chloride, prior to siYY2/ siMOCK and spermidine complex formation using an 8:1 molar ratio of the polyamine nitrogen to nucleotide phosphate. One hundred nanomoles of siYY2 or siMOCK per 100 mg of polymer in Tris‐EDTA (10 mmol/L Tris‐HCl and 1 mmol/L EDTA) buffer (Sigma‐Aldrich) were added dropwise to the PLGA solution while vortexing. This solution was sonicated and subsequently added to 2.5% polyvinyl alcohol and a 5 mg/mL avidin‐palmitate solution for the second emulsion. The nanoparticles were hardened during solvent evaporation in 0.3% polyvinyl alcohol for 3 hours. To synthesize unmodified nanoparticles, the second emulsion contained only 2.5% polyvinyl alcohol, and nanoparticles were incubated post‐hardening in PBS without ligand for 30 minutes. All of the nanoparticles were washed twice in deionized water to remove residual solvent, centrifuged at 4℃, lyophilized, and stored at −20℃. As previously reported,^13^, ^21^, ^22^, ^23^ 5 mg of siRNA@PLGA was dissolved in 0.5 mL of methylene chloride for 30 minutes, and siYY2/siMOCK was extracted twice into Tris‐EDTA buffer. Encapsulation efficiency was determined by comparing the amount of siRNA loaded onto the PLGA nanoparticles with a theoretical loading of 1 nmol siRNA/mg polymer. For the siRNA@PLGA (nanoparticle‐siRNA‐CH2.5), the loading was 514 pmol siRNA/mg nanoparticle. The administered dosage of siRNA@PLGA was 400 μL (20 mg/mL) once every 2 days.

### 2.3 Establishment of cDNA sequencing libraries and high‐throughput RNA‐Seq

The following analysis was executed by KangChen Biotech. In accordance with their experimental procedures, a random fragment sequencing library was constructed using a SOLiD Whole Transcriptome Analysis Kit (Life Technologies). Nucleic acid cleaving reagents were added, and the mRNA was randomly disrupted into short segments in a shaking incubator. First‐strand cDNA was reverse transcribed using the fragmented mRNA as the template; and second‐strand cDNA was synthesized using a second‐strand DNA‐synthesis reaction system consisting of DNA polymerase I, dNTPs, and RNase H (Sigma). The synthesized DNA was purified using a DNA purification kit and recovered. The base ‘A’ was added to the 3’end of the cDNA, followed by ligation to the adapter in order to complete the blunt‐end repair reaction. Subsequently, we performed DNA fragment‐size selection. Finally, the cDNA was used for PCR amplification to obtain a sequencing library. The constructed library was qualified using an Agilent 2100 Bioanalyzer and the ABI StepOnePlus Real‐Time PCR System, and it was subjected to high‐throughput sequencing using an Illumina HiSeq™ 2000 Sequencer after passing quality controls.

### 2.4 Statistical analysis

Each experiment was performed at least three times, and data are presented as the mean ± standard error, where applicable. Differences were evaluated with the Student's *t* test. A *P* value less than .05 was considered to be statistically significant.

## 3 RESULTS

### 3.1 Adenosine thymopentin significantly improves POF syndrome induced by HFHS in mice

**FIGURE 1:**
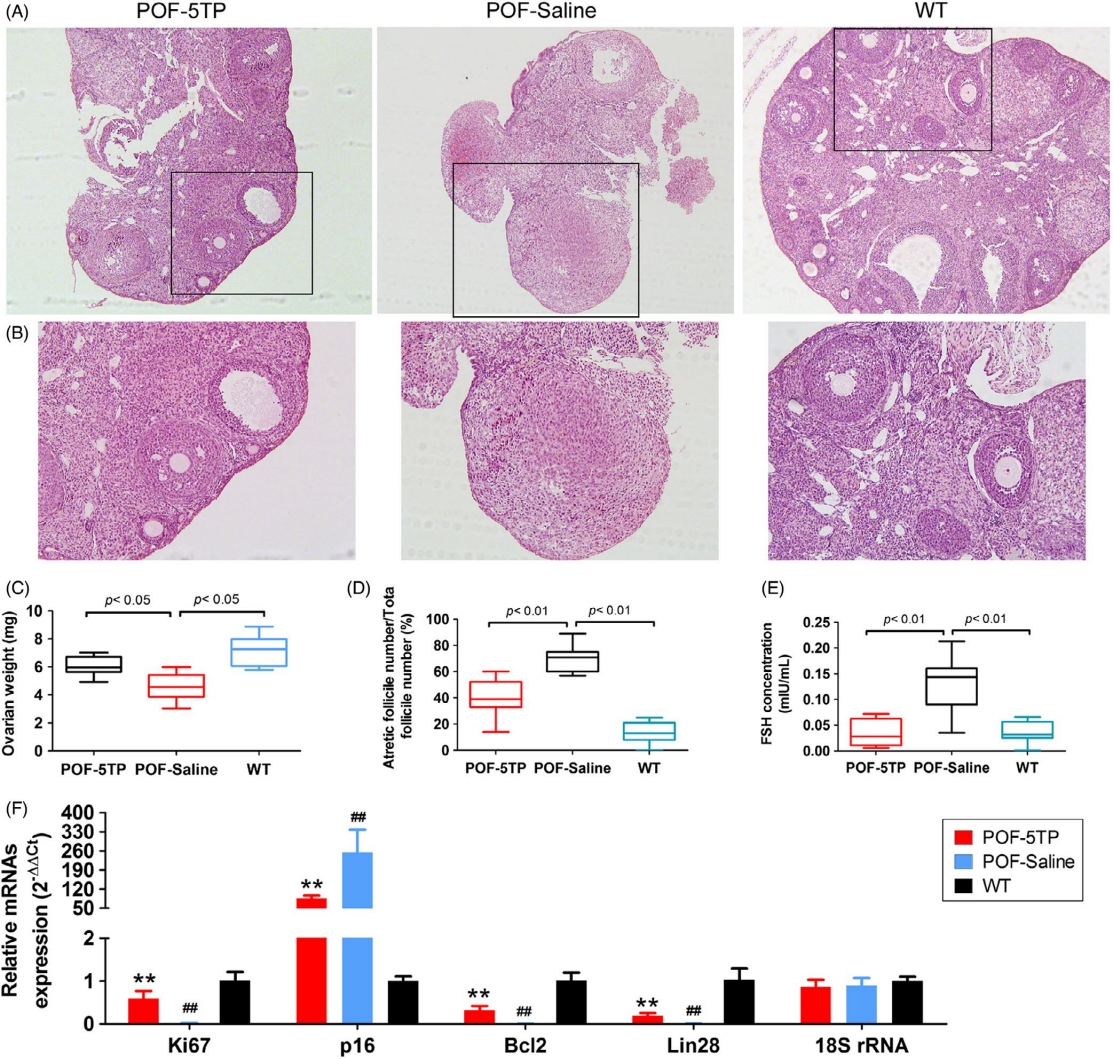
TP‐5 significantly improves the symptoms of POF induced by HFHS in mice. A, Pathologic examination of the ovary under low‐power microscopy. Each bottom panel is the magnified black box in the upper panel under high power (100×). B, The results of ovarian pathology under high‐power microscopy (magnification 200×). C, Ovarian weights. D, The ratio of atretic to total follicles. F, FSH levels in peripheral blood of mice in each group. G, QPCR was used to determine the markers of cellular proliferation and ageing. **P < .01 vs POF‐Saline group, ^##^ *P* < .01 vs WT group, *t* test, n = 4

**FIGURE 2:**
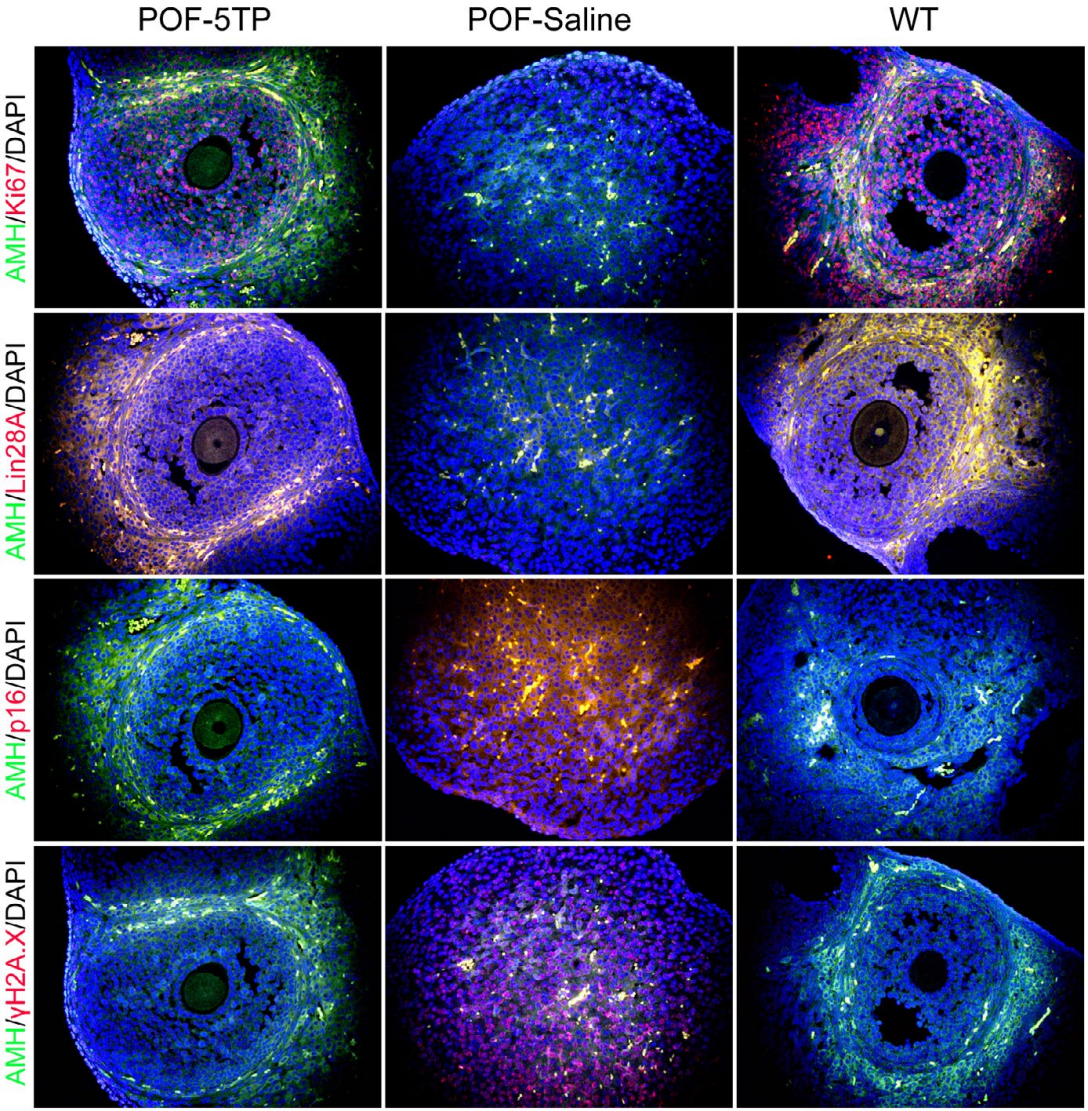
Immunofluorescence staining results of markers of cellular proliferation and ageing

Histopathologic examination of each group of mouse ovaries showed few follicles of any developmental stage in the HFHS group (POF‐Saline), while multiple mature oocytes and follicles were found in the ovaries of normal mice in the WT group. The incidence of atretic follicles in the ovaries from 5TP‐treated mice, however, was significantly improved (Figure 1A,B), and 5TP significantly increased ovarian weights (Figure 1C) of HFHS mice. The results of follicle counting showed that the proportion of atretic follicles in the POF group was significantly elevated relative to the WT group, while the proportion of atretic follicles in the 5TP group was significantly lower than in the POF group (Figure 1D). HFSH augmented the concentrations of FSH in peripheral blood of POF mice compared with FSH in the WT group, while 5TP attenuated the levels of FSH in POF mice (Figure 1E). Thus, our experimental results indicated that TP‐5 can significantly improve the symptoms of POF. In addition, qPCR and immunofluorescence staining showed that 5TP significantly enhanced the expression of Ki67, Bcl2 and Lin28 (a marker of ovarian granulosa cell proliferation) and diminished the expression of p16 and γ H2A.X (Figures 1F,2) in ovarian granulosa cells of mice after induction of POF by HFHS. Therefore, thymopentin significantly ameliorated ageing of ovarian granulosa cells and POF symptoms induced by HFHS in mice.

### 3.2 Thymopentin modifies the differential expression of multiple transcription factors in ovarian tissue

**FIGURE 3:**
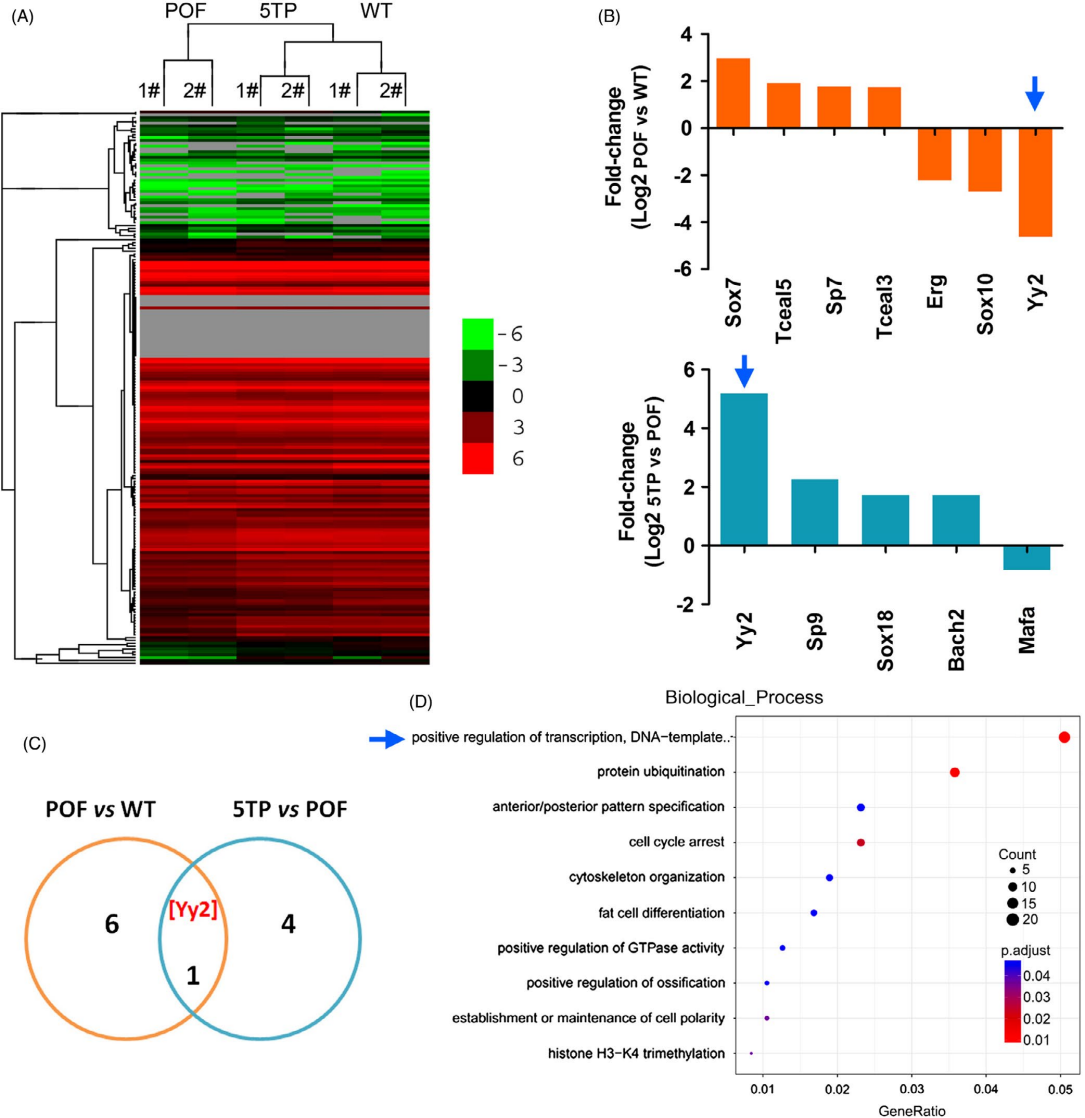
TP‐5 alters the differential expression of multiple transcription factors in ovarian tissue. A, Heatmap analysis revealed differences in the expression of multiple transcription factors among the groups. B, TP‐5 treatment significantly induced the expression of multiple transcription factors. C, Statistical analysis showed that the differential changes in transcription factor YY2 involved all groups. D, GO analysis indicated the enrichment prediction results of differentially expressed transcription factors in biological process

We performed RNA‐Seq transcriptome sequencing on 6 samples of mouse ovarian tissue from HFHS‐5TP (5TP), HFHS‐Saline (POF) and WT groups, and analysed all of the known transcription factors in depth. The RNA‐Seq analysis showed that there were differences in the expression of transcription factors among the three groups (Figure 3A, Table S3). Compared with the WT group, 7 transcription factors in the POF group had significant changes in mRNA expression of which 4 were significantly upregulated (log2 (POF/WT) > 1.8 and *P* < .01) and 3 were significantly downregulated (log2 (POF/WT) <−2 and *P* < .01) (Figure 3B). Compared with the POF group, the mRNA expression of 5 transcription factors in the 5TP group changed significantly of which 4 (Yy2, Sp9, Sox18 and Bach2) were significantly upregulated and 1 (Mafa) was significantly downregulated. We also observed a difference in the expression of the transcription factor Yinyang2 (Yy2) across the three samples (Figure 3C). The results of Kyoto Encyclopedia of Genes (KEGG) analysis showed that the expression difference of genes covered by the positive regulation of transcription was the most significant between the two groups (Figure 3D). Our results thus showed that thymopentin altered the differential expression of many transcription factors in ovarian tissue, transcription factor Yy2 in particular.

### 3.3 YY2 promotes transcriptional activation by binding to specific sites of the Lin28A promoter

**FIGURE 4:**
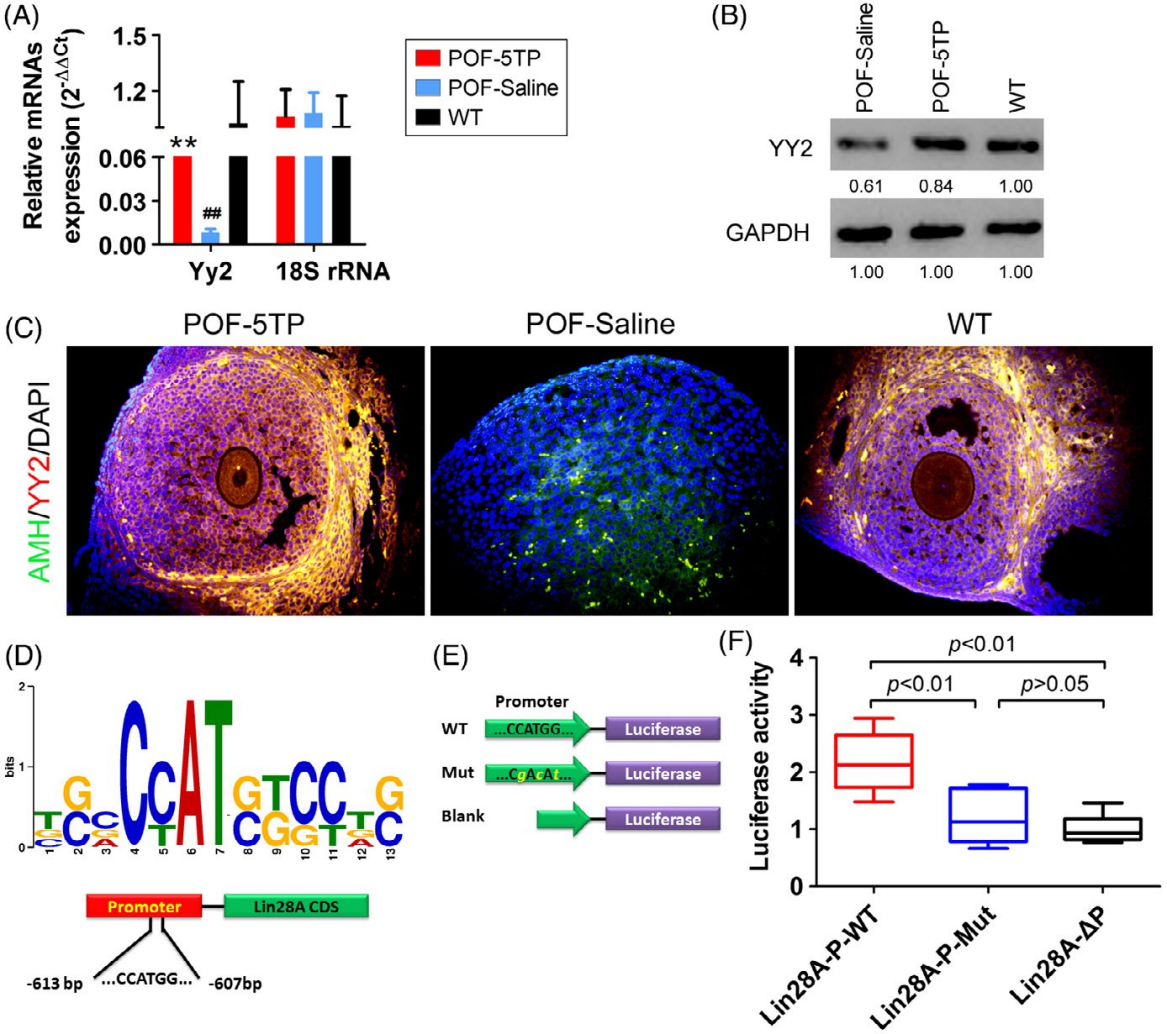
YY2 promotes its transcriptional activation by binding to specific sites on the Lin28A gene promoter. A, The results of qPCR detection of Yy2 expression levels in ovarian tissues from each group. **P < .01 vs POF‐Saline group, ^##^ *P* < .01 vs WT group, *t* test, n = 4. B, Western blotting analysis of the expression levels of Yy2 in ovarian tissues of each group. C, Immunofluorescence staining of the expression of Yy2 in ovarian tissues from each group. D, Bioinformatics analysis predicted that the recognition site of YY2 was C(C/T)AT(G/C)(G/T). E, The cloning site of the luciferase reporter plasmid promoter contains the YY2 recognition sequence. F, Luciferase reporter assay results

Both qPCR and Western blotting results indicated that HFHS significantly decreased the expression of YY2 in mOGCs, while 5TP reversed the above phenomena (Figure 4A,B; Figure S1), and the results of tissue immunofluorescence staining were consistent with the above experimental results (Figure 4C). Considering that YY2 is a typical transcription factor, we referred to previous reports ^24^, ^25^, ^26^, ^27^, ^28^, ^29^, ^30^ and used bioinformatics tools to analyse the promoter sequence of the Lin28A gene in order to find the binding site between YY2 and the Lin28A promoter. The predicted results suggested that there is a potential binding site of YY2 at the −613 bp to the −607 bp positions (Figure 4D) of the Lin28A gene promoter and that its sequence is C(C/T)AT(G/C)(G/T). After luciferase reporter assay verification, we found that luciferase activity was significantly increased when the reporter plasmid carried the sequence of YY2 binding sites, while when the reporter plasmid carried mutation sites or empty plasmids, the activity decreased significantly (Figure 4E,F). Therefore, these results showed that YY2 promotes transcriptional activation of the Lin28A gene by binding to specific sites on its promoter.

### 3.4 Silencing the expression of endogenous YY2 weakens the therapeutic effect of thymopentin on POF mice

**FIGURE 5:**
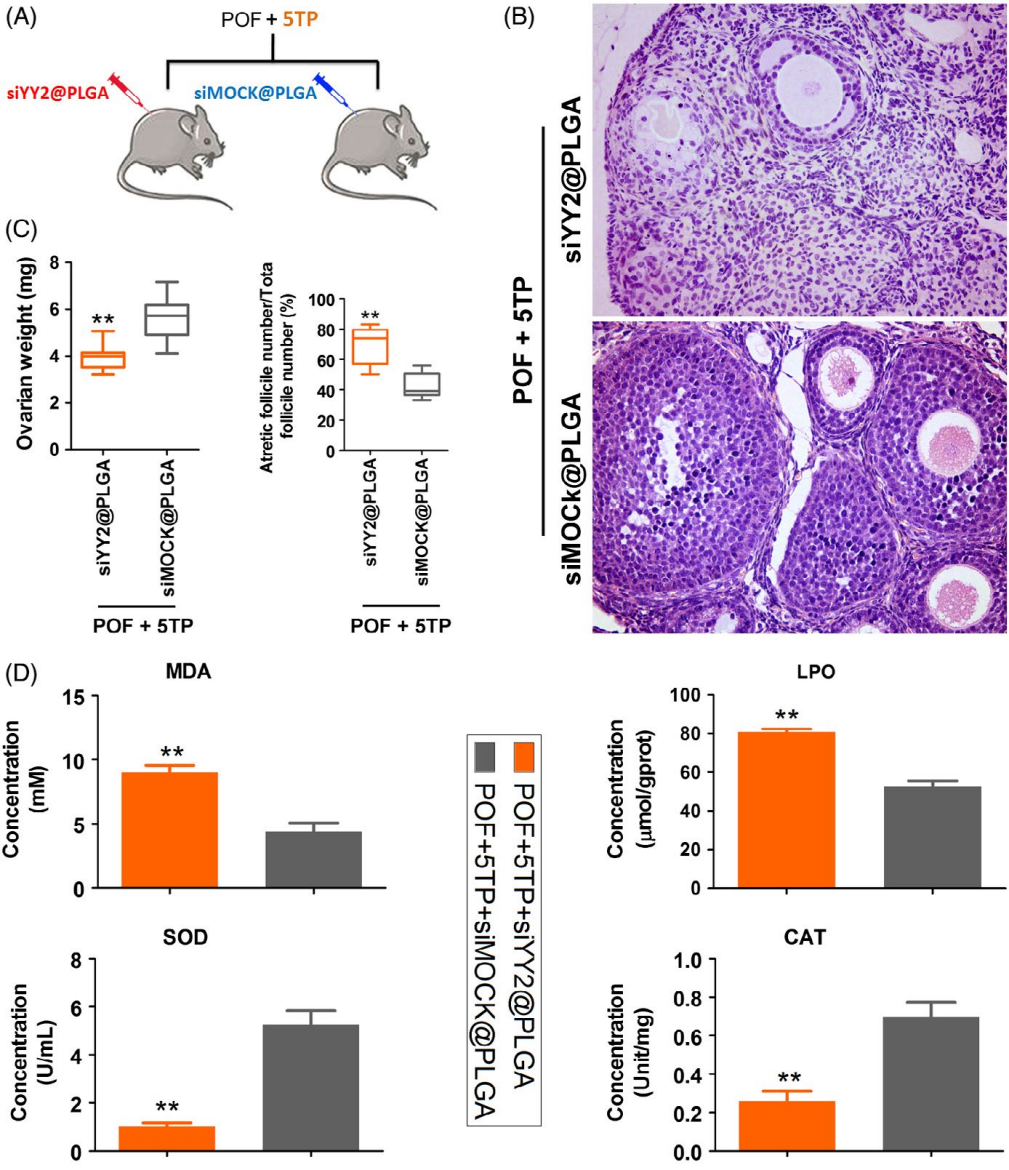
Silencing the expression of endogenous YY2 weakens the therapeutic effects of TP‐5 on POF mice. A, Diagrammatic representation of groups. B, Ovarian pathology was identified with haematoxylin and eosin staining (magnification, 200×). C, Ovarian weight was lower in the POF‐5TP‐siMock@PLGA group than in the POF‐5TP‐siMock@PLGA group. More atretic follicles were present in the POF‐5TP‐siYY2@PLGA group mice than in the POF‐5TP‐siMock@PLGA group. **P < .01 vs POF‐5TP‐siMock@PLGA group, *t* test, n = 8. D, The content of MDA and LPO was significantly higher in the ovaries of POF‐5TP‐siYY2@PLGA group mice than in the ovaries of POF‐5TP‐siMock@PLGA group mice. SOD and CAT activities were significantly lower in the ovaries of POF‐5TP‐siYY2@PLGA group mice than in the ovaries of POF‐5TP‐siMock@PLGA group mice. **P < .01 vs. POF‐5TP‐siMock@PLGA group, *t* test, n = 4

**FIGURE 6:**
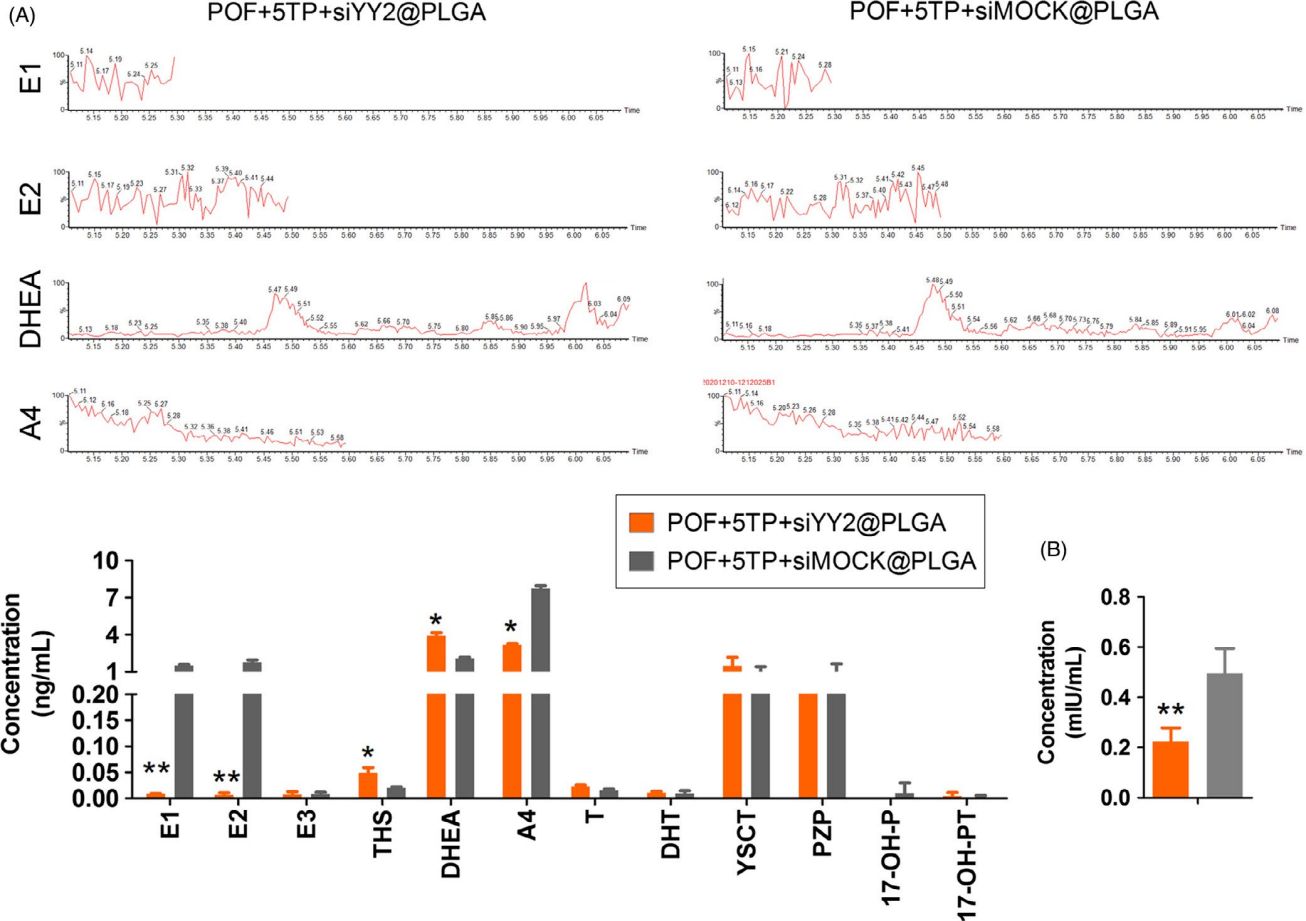
Steroid hormone and FSH levels in mice as evaluated by HPLC‐MS/MS. A, The steroid hormone levels in mice as evaluated by HPLC‐MS/MS. E1, oestrone; E2, oestradiol; THS, melatonin; DHET, dehydroepiandrosterone; A4, androstenedione. **P < .01 vs POF‐5TP‐siMock@PLGA group,*P < .05 vs POF‐5TP‐siMock@PLGA group, *t* test, n = 4. B, The FSH level in mice as evaluated by HPLC‐MS/MS. **P < .01 vs POF‐5TP‐siMock@PLGA group, *t* test, n = 4

**FIGURE 7:**
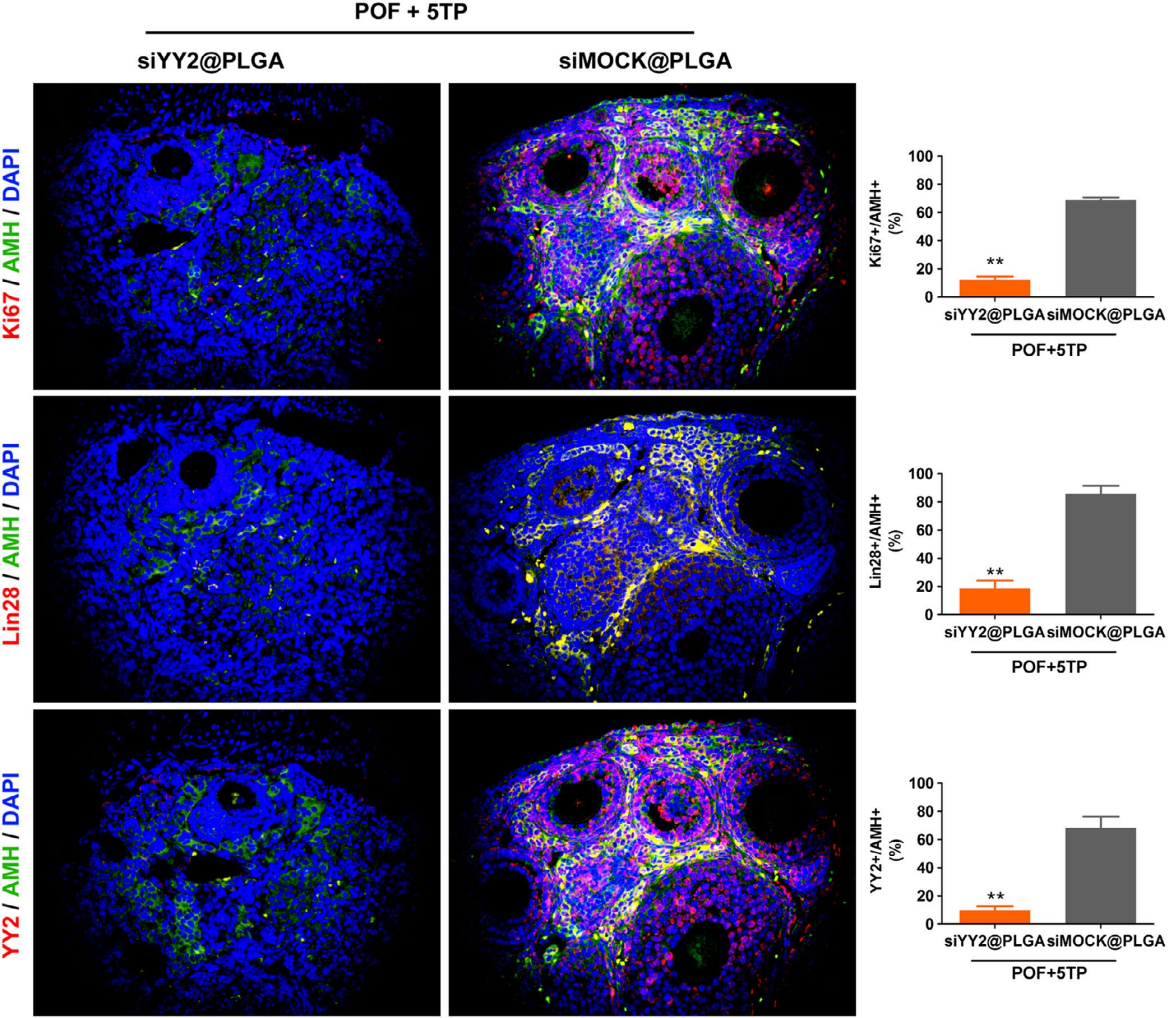
Immunofluorescence staining results of cell proliferation markers and YY2

**FIGURE 8:**
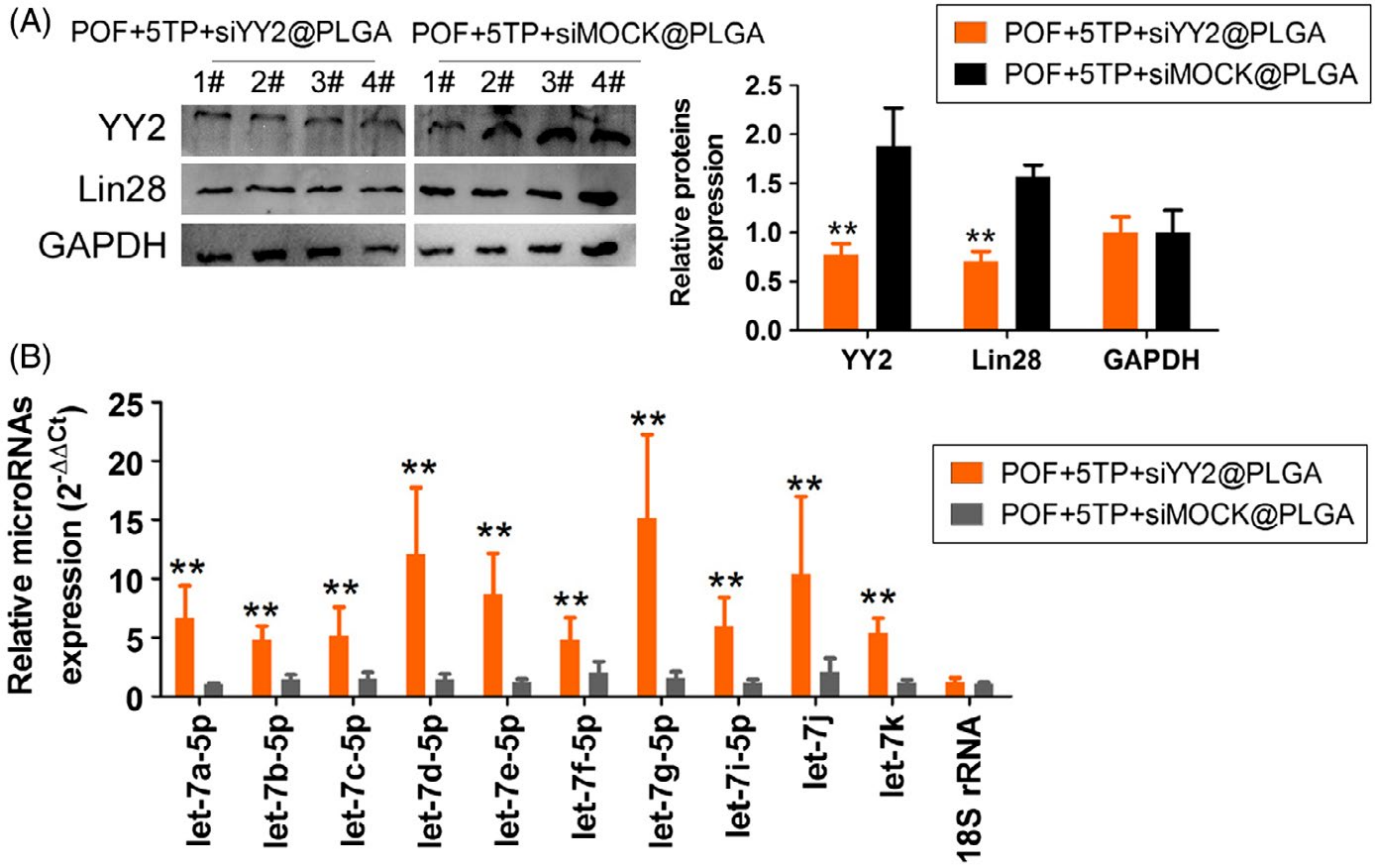
The expression of let‐7 microRNAs is induced by decreasing the expression of endogenous YY2. A, Western blotting analysis results. **P < .01 vs POF‐5TP‐siMock@PLGA group, *t* test, n = 4. B, Let‐7microRNAs expression level using qPCR. **P < .01 vs POF‐5TP‐siMock@PLGA group, *t* test, n = 4

To study the relationship between YY2/Lin28A and thymopentin, siYY2@PLGA was injected intraperitoneally into mice, and 5TP was combined with HFHS intervention (Figure 5A). One month later, we found that the ovarian weights in the siYY2@PLGA injection group were significantly reduced relative to those in the siMOCK@PLGA injection group, and the results from counting all types of follicles showed that the number of normal follicles in the siYY2@PLGA injection group was significantly lower than that in the siMOCK@PLGA injection group, while the number of atretic follicles was higher (Figure 5B,C). Besides, the content of MDA and LPO was significantly higher in the ovaries of siYY2@PLGA group mice than in the ovaries of siMock@PLGA injection group (Figure 5D). SOD and CAT activities were significantly lower in the ovaries of siYY2@PLGA group mice than in the ovaries of siMock@PLGA injection group (Figure 5D). The results of steroid hormone determinations by HPLC‐MS/MS showed that the levels of the oestrogens oestrone (E1) and oestradiol (E2) in peripheral blood of mice injected with siYY2@PLGA were significantly lower than for mice injected with siMOCK@PLGA, while the levels of hormones such as melatonin (THS), dehydroepiandrosterone (DHEA) and androstenedione (A4) were elevated (Figure 6). Tissue immunofluorescence staining and Western blotting results showed that the protein expression of YY2, Lin28 and Ki67 in ovarian granulosa cells in the siYY2@PLGA injection group was significantly lower than in the siMOCK@PLGA injection group (Figures 7,8A). However, qPCR results showed that the expression level of let‐7 family microRNAs in ovarian granulosa cells in the siYY2@PLGA injection group was significantly higher than that in the siMOCK@PLGA injection group (Figure 8B). The above results suggested that silencing the expression of endogenous YY2 weakened the therapeutic effect of thymopentin on POF mice, primarily because the downregulation of YY2 expression led to a decrease in Lin28A transcriptional activity, upregulation of the expression of let‐7 family microRNAs and inhibition of the division and proliferation of ovarian granulosa cells.

### 3.5 Exosomal Yy2 is a potential serum biomarker for the diagnosis of patients with POF

**FIGURE 9:**
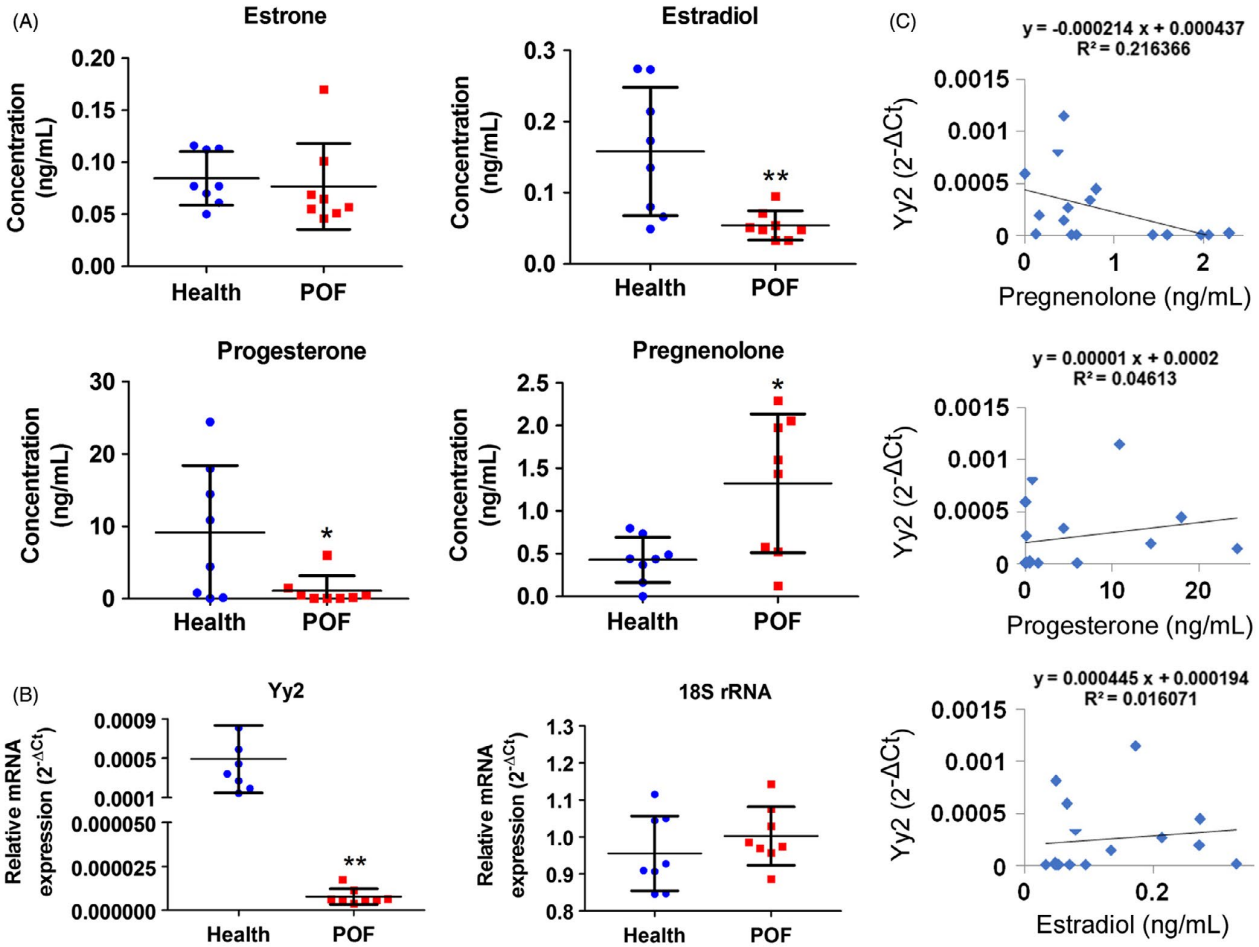
Exosomal YY2 constitutes a potential biomarker in blood for the diagnosis of POF patients. A, The results of hormone levels in human peripheral blood**P < .01 vs healthy group, *P < .05 vs healthy group, *t* test, n = 8. B, The expression of exosome‐derived YY2 mRNA in human peripheral blood was detected by qPCR**P < .01 vs healthy group, *t* test, n = 8. C, Correlation analysis of peripheral blood hormone levels and the expression levels of exosome‐derived YY2 mRNA in human peripheral blood

We measured the levels of steroid hormones in peripheral blood from 8 patients with premature ovarian failure and 8 women with normal ovarian function and uncovered an augmented level of pregnenolone in patients with POF (Figure 9A), while the levels of progesterone and oestradiol were diminished (Figure 9A). Subsequently, we isolated peripheral blood exosomes from patients with POF and healthy women, and our qPCR results revealed that the Yy2 mRNA level in exosomes derived from peripheral blood from POF patients was significantly reduced compared to healthy women (Figure 9B). These results were therefore in good agreement with those involving the POF mouse model. When we subsequently compared the statistical correlation between hormone levels and Yy2, we noted a significant negative correlation between pregnenolone and Yy2, while there was a significant positive correlation between the expression levels of progesterone/oestradiol and Yy2 (Figure 9C). As Yy2 in peripheral blood‐derived exosomes displayed a specific correlation with the course of disease and hormonal levels in POF, Yy2 constitutes a potential diagnostic marker.

## 4 DISCUSSION

Premature ovarian failure is a type of reproductive endocrine disorder that in a broad sense is manifested as pathologic ageing of the reproductive system.^2^, ^3^, ^4^, ^5^, ^31^ There are many pathogenic factors of POF, including heredity, immunity and inflammation, metabolic and endocrine disorders, mental illness and neurotransmitter disorders. Investigators have increasingly focussed on the adverse effects of high‐fat and high‐sugar (HFHS) diets in recent years. It is postulated that HFHS increases the risk of obesity, tumours and cardiovascular disease,^7^, ^8^, ^9^ and seriously affects ovarian function and oocyte quality.^10^, ^11^, ^12^ Our recent studies have further confirmed some epigenetic mechanisms that underly ovarian granulosa cell ageing and premature ovarian failure due to an HFHS diet, including an inhibition of endogenous miR‐146, activation of the DAB2IP/ASK1/p38‐signalling pathway and induction of γ‐H2A.X phosphorylation.^13^ All of these studies suggested that metabolic disorders induced by HFHS increased the risk for POF.

Thymopentin (5TP) is a biochemical preparation that improves immunity and anti‐ageing.^14^, ^15^, ^16^, ^17^, ^18^ It is typically used to enhance the immunity of the elderly with low immune function or to improve impaired immune function after radiotherapy and chemotherapy in patients with malignant tumours.^14^, ^15^, ^16^, ^17^, ^18^ Although 5TP is known to effectively regulate the activity and homeostasis of immune cells and the release of immune factors,^14^, ^15^, ^16^, ^17^, ^18^ there are no reports of its effects on POF. Previous studies have shown that one of the functions of 5TP is to induce T‐cell differentiation by selectively inducing the transformation of Thy‐1‐prothymocytes into Thy‐1 + T cells,^14^, ^15^, ^16^, ^17^, ^18^ which involves the mediation of increased cAMP. Another essential function of thymopentin is the specific receptor binding to mature peripheral blood T cells, which increases the levels of intracellular cAMP and induces a series of intracellular responses that form the basis of its immunomodulatory function.^14^, ^15^, ^16^, ^17^, ^18^ Under normal conditions, 5TP displays an immunostimulatory effect in which splenic lymphocytes undergo significantly increased rates of E rosette formation and transformation. 5TP also regulates the polarity of immune cells and stimulates the release of inflammatory factors at different stages of the immune response. Since 5TP exerts typical anti‐ageing and regulatory immune effects, we attempted to assess its therapeutic effect on the POF mouse model. Surprisingly, 5TP significantly ameliorated follicular atresia and hormonal disorders induced by HFHS in POF mice, significantly inhibiting the deposition of lipid peroxides, thus weakening the damaging effects of oxidative stress. Thus, this study provides a new direction for the treatment of POF and the novel use of a relatively old drug, 5TP. We uncovered no previous reports that suggest that 5TP can treat POF, but our experimental data confirmed that it has a therapeutic effect on POF.

YY2 is the most recently described member of the Yin Yang family of transcription factors.^32^ Despite its high sequence identity and functional similarity to the well‐characterized YY1, less is known about YY2’s role in biologic processes.^32^ It is currently understood that YY2 plays an important role in the regulation of mouse embryonic development and tumour metastasis; however, its regulation of ovarian function has not been investigated. We herein first confirmed a positive correlation between ovarian granulosa cell status and the expression level of YY2 protein, which indicated that YY2 is essential for the maintenance of ovarian function. In addition, we uncovered a potentially novel DNA‐binding site for YY2. According to previous reports, the binding sites of YY2 to the target gene promoter are principally the following: 5′‐(A/c/g)(A/t)NATG(G/a/t)(C/a)(G/c/t)‐3′,^27^, ^28^ 5′‐CGCCATNTT‐3′,^27^ 5′‐A/GAA/GG/ATGG(C)‐3′, and 5′‐ANAGAAGTGG‐3′.^33^ Based upon previously existing research data and data predicted by bioinformatics tools, we found that YY2 recognizes the specific nucleic acid site sequence of the target gene promoter 5'‐C(C/T)AT(G/C)(G/T)‐3'. These investigators ascertained that YY2 protein manifests flexibility and diversity in recognizing DNA sequences and that it does not possess a single recognition site like some transcription factors or DNA‐binding proteins.

In addition, the present study also contained a significant innovation: the use of poly (lactic‐*co*‐glycolic acid) (PLGA) nanoparticles as a vector to achieve the transfection and expression of siRNA oligos in mice in vivo. PLGA is prepared by random polymerization of lactic acid and glycolic acid. It possesses adequate biocompatibility, is not toxic, has favourable properties of encapsulation and film formation, and is widely used in pharmaceutical, medical engineering and modern industrial fields.^19^, ^20^, ^34^ As such, it is a biodegradable and functional polymeric organic compound. The degradation products of PLGA are lactic acid and glycolic acid, both of which are by‐products of human metabolic pathways, and thus, there are not expected to be any toxic side effects (except for lactose deficiency) when PLGA is used in nano‐drug delivery systems.^19^, ^20^, ^34^ PLGA has been widely used as a nano‐medical material in skin transplantation, wound suture, in vivo implantation, micro‐nanoparticles and other fields.^19^, ^20^, ^34^ As a nano‐carrier, PLGA has been widely adapted to preclinical studies of tumour therapy.^19^, ^20^, ^34^ Zhu et al used D‐α‐tocopherol polyethylene glycol succinate (TPGS) as a pore‐forming agent to prepare porous PLGA nanoparticles that entail a nano‐precipitation method to deliver docetaxel and TPGS to HeLa cells and a xenograft tumour model in vivo. Cytotoxicity analysis and use of xenograft tumour models showed that DTX/TPGS‐loaded porous PLGA nanoparticles had a more obvious anti‐tumour effect and that the inhibitory effect on HeLa cells was greater than that of PLGA nanoparticles without TPGS, the former inhibited multidrug resistance and enhanced the overall anti‐tumour effect.^34^ Martin et al^21^ used chitosan‐modified PLGA nanoparticles to target a silencing siRNA oligoRNA (siGP130@PLGA) of GP130 and injected it into a mouse model of bladder cancer xenotransplantation; these authors achieved the goal of over‐expression of siRNA in vivo, thus significantly downregulating endogenous GP130.^21^ Their experimental results suggested that siGP130@PLGA injection reduced tumour volume by approximately 70% compared with the control group.^21^ In our study, we adopted the methodology of Martin et al and used chitosan‐functionalized PLGA nanoparticles to link the siRNA oligo to target YY2 to evaluate the function of siYY2@PLGA in vivo and in vitro. Our results showed that PLGA could successfully transport and express siRNA in ovarian granulosa cells, with the siYY2@PLGA complex retaining the high activity of RNAi in vivo. This method thus ultimately and effectively silenced the expression of the target gene *Yy2*, further elucidating the carrier function of PLGA nanoparticles and confirming the delivery of siRNA in vivo.

On the other hand, in this study, we found that the expression level of YY2 gene in murine ovarian granulosa cells was affected by high glucose and high fat through high‐throughput sequencing. And, thymopentin could significantly stimulate the expression level of YY2 in mOGCs from POF mice. This phenomenon aroused our strong interest. We finally found that thymopentin alleviated murine POF symptom by activating YY2/Lin28A signalling pathway and inhibiting the expression of let‐7 family microRNAs in mOGCs through in vitro and in vivo studies. However, considering that there are few studies on the regulation of gene expression by thymopentin, in this study, we did not explore how it activates YY2 expression. There are various evidences reveal that the expression of YY2 gene activated by thymopentin is located at the level of gene transcription (because the mRNA of YY2 gene is highly expressed in the murine ovarian tissue and ovarian granulosa cells of thymopentin treated mice, indicating that it has high transcriptional activity). According to the current reports, there are many ways to activate a gene transcription, such as demethylation of CpG island on gene promoter and acetylation modification of histone specific sites. Therefore, further study on which pathway thymopentin activates the transcriptional activity of YY2 gene will be the focus of our next project.

**FIGURE 10:**
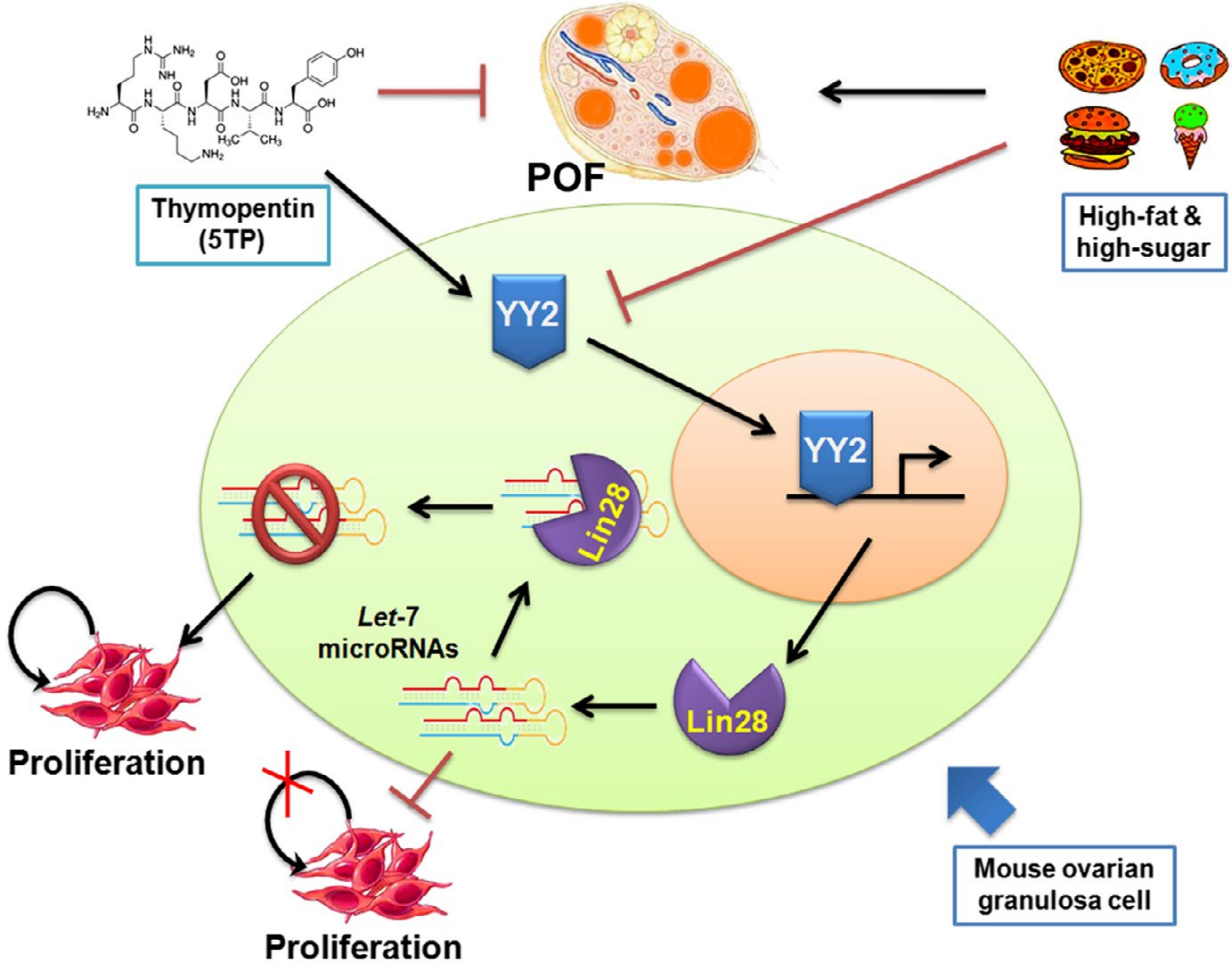
TP‐5 regulates the expression of the YY2/Lin28/let‐7 microRNA‐signalling pathway axis to achieve a therapeutic effect on HFHS‐POF

With this study, we fully confirmed the use of thymopentin to alleviate the ageing of ovarian granulosa cells by activating the expression of transcription factor YY2 in ovarian granulosa cells. Thymopentin promoted the transcriptional activation of Lin28A and inhibited the activity of microRNAs of the let‐7 family, ultimately achieving a therapeutic effect on POF in mice (Figure 10). Therefore, YY2 derived from human peripheral blood exosomes can also be used as a potential biomarker to evaluate the occurrence and development of POF.

## CONFLICT OF INTEREST

We declared no potential conflicts of interest.

## AUTHORS' CONTRIBUTIONS

Te Liu, Fangyuan Jing, Peirong Huang, Zixiang Geng performed the majority of the experiments in the study. Jianghong Xu, Jiahui Li, Danping Chen, Ying Zhu, Zhenxin Wang contributed to the analysis of experimental data. Te Liu, Willian Huang, Chuan Chen contributed to the study design, manuscript writing and provided experimental funding support. All authors read and approved the final manuscript.

## Supporting information

Figure S1

Table S1

Table S2

Table S3

Supplementary Material

## Data Availability

The data that support the findings of this study are available from the corresponding author upon reasonable request.
