# Supplementary material for: Thymopentin alleviates premature ovarian failure in mice by activating YY2/Lin28A and inhibiting the expression of let‐7 family microRNAs
Source: Cell Prolif. 2021 Jun 28;54(8):e13089. doi: 10.1111/cpr.13089 (PMC8349654; doi:10.1111/cpr.13089)
Supplement: Supplementary file 1 — Figure S1 [file CPR-54-e13089-s004.docx]

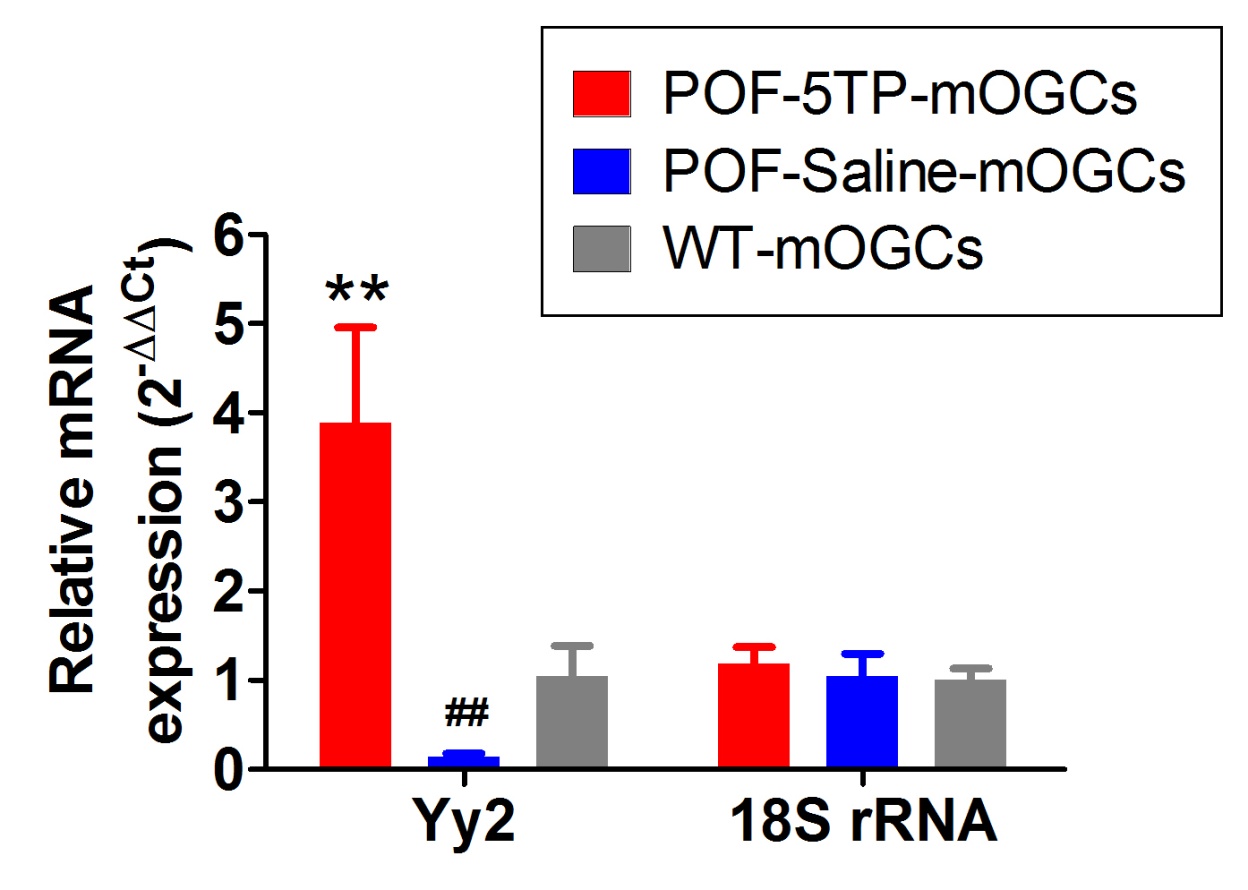


**Figure S1. The 5TP promotes YY2 expression in murine primary ovarian granulosa cells (mOGCs).** The results of qPCR detection of Yy2 expression levels in mOGCs from each group. **p < 0.01 vs. POF-Saline group, ##p < 0.01 vs. WT group, *t*-test, n = 4.
