# Supplementary material for: Thymopentin alleviates premature ovarian failure in mice by activating YY2/Lin28A and inhibiting the expression of let‐7 family microRNAs
Source: Cell Prolif. 2021 Jun 28;54(8):e13089. doi: 10.1111/cpr.13089 (PMC8349654; doi:10.1111/cpr.13089)
Supplement: Supplementary file 2 — Table S1 [file CPR-54-e13089-s002.docx]

**Table S1 The PCR Primers list**

| **Primer Name** | **Sequences (5' to 3')** |
| --- | --- |
| Human YY2-F | ATGGCCTCCAACGAAGATTTC |
| Human YY2-R | TCCGTCGGAATGTCCTCCATA |
| Mouse YY2-F | TGCTGCCACCGGATAATATCG |
| Mouse YY2-R | GCCCAATCGACACCTACATCT |
| Human Lin28A-F | TGCGGGCATCTGTAAGTGG |
| Human Lin28A-R | GGAACCCTTCCATGTGCAG |
| Mouse Lin28A-F | GGCATCTGTAAGTGGTTCAACG |
| Mouse Lin28A-R | GCCAGTGACACGGATGGATT |
| Human Lin28B-F | AAGAAGTGCCATTACTGTCAGAG |
| Human Lin28B-R | GCCTCCTGAGGAAACGGTG |
| Mouse Lin28B-F | AGAATGCAGTCTACCTCCTCAG |
| Mouse Lin28B-R | CCTCCCACTTCTCTTGGTGC |
| mmt-let-7a-5p-F | TGAGGTAGTAGGTTGTATAGTT |
| mmt-let-7a-5p-R | GCTGTCAACGATACGCTACCTA |
| mmt-let-7b-5p-F | TGAGGTAGTAGGTTGTGTGGTT |
| mmt-let-7b-5p-R | GCTGTCAACGATACGCTACCTA |
| mmt-let-7c-5p-F | TGAGGTAGTAGGTTGTATGGTT |
| mmt-let-7c-5p-R | GCTGTCAACGATACGCTACCTA |
| mmt-let-7d-5p-F | AGAGGTAGTAGGTTGCATAGTT |
| mmt-let-7d-5p-R | GCTGTCAACGATACGCTACCTA |
| mmt-let-7e-5p-F | TGAGGTAGGAGGTTGTATAGTT |
| mmt-let-7e-5p-R | GCTGTCAACGATACGCTACCTA |
| mmt-let-7f-5p-F | TGAGGTAGTAGATTGTATAGTT |
| mmt-let-7f-5p-R | GCTGTCAACGATACGCTACCTA |
| mmt-let-7g-5p-F | TGAGGTAGTAGTTTGTACAGTT |
| mmt-let-7g-5p-R | GCTGTCAACGATACGCTACCTA |
| mmt-let-7i-5p-F | TGAGGTAGTAGTTTGTGCTGTT |
| mmt-let-7i-5p-R | GCTGTCAACGATACGCTACCTA |
| mmt-let-7j-F | TGAGGTATTAGTTTGTGCTGTTAT |
| mmt-let-7j-R | GCTGTCAACGATACGCTACCTA |
| mmt-let-7k-F | TGAGGTAGGAGGTTGTGTG |
| mmt-let-7k-R | GCTGTCAACGATACGCTACCTA |
| Human 18SrRNA-F | CAGCCACCCGAGATTGAGCA |
| Human 18SrRNA-R | TAGTAGCGACGGGCGGTGTG |
| Mouse 18SrRNA-F | AGGGGAGAGCGGGTAAGAGA |
| Mouse 18SrRNA-R | GGACAGGACTAGGCGGAACA |
