# Supplementary material for: Thymopentin alleviates premature ovarian failure in mice by activating YY2/Lin28A and inhibiting the expression of let‐7 family microRNAs
Source: Cell Prolif. 2021 Jun 28;54(8):e13089. doi: 10.1111/cpr.13089 (PMC8349654; doi:10.1111/cpr.13089)
Supplement: Supplementary file 3 — Table S2 [file CPR-54-e13089-s003.docx]

**Table S2 The antibodies list**

| **Antibodies** | **Companies** | **Applications** |
| --- | --- | --- |
| Mouse anti-AMH antibody [5/6] (ab24542) | Abcam, MA, USA | IF (1:300) |
| Rabbit anti-Ki-67 (D3B5) antibody (#9129) | Cell Signaling Technology, MA, USA | IF (1:300) |
| Rabbit anti-LIN28A (D1A1A) XP® antibody (#8641) | Cell Signaling Technology, MA, USA | IF (1:300) |
| Rabbit anti-Histone H2A.X (D17A3) XP® antibody (#7631) | Cell Signaling Technology, MA, USA | IF (1:300) |
| Rabbit anti-CDKN2A/p16INK4a antibody [EPR20418] (ab211542) | Abcam, MA, USA | IF (1:300) |
| Rabbit anti-YY2 antibody (A-5) (sc-377008) | Santa Cruz Biotechnology, MA, USA | IF (1:300)  WB (1:1000) |
| Rabbit anti-GAPDH antibody [EPR16891] (ab181602) | Abcam, MA, USA | WB (1:1000) |
| Goat anti-Rabbit IgG H&L (HRP) (ab97051) | Abcam, MA, USA | WB (1:1000) |
| Goat anti-Mouse IgG H&L (HRP) (ab6789) | Abcam, MA, USA | WB (1:1000) |
| Goat anti-Mouse IgG H&L (Alexa Fluor® 488) (ab150113) | Abcam, MA, USA | IF (1:300) |
| Goat anti-Rabbit IgG H&L (Alexa Fluor® 555) (ab150078) | Abcam, MA, USA | IF (1:300) |
