# Supplementary material for: Thymopentin alleviates premature ovarian failure in mice by activating YY2/Lin28A and inhibiting the expression of let‐7 family microRNAs
Source: Cell Prolif. 2021 Jun 28;54(8):e13089. doi: 10.1111/cpr.13089 (PMC8349654; doi:10.1111/cpr.13089)
Supplement: Supplementary file 4 — Table S3 [file CPR-54-e13089-s005.docx]

**Table S3 The results of RNA-Seq**

| **#ID** | **Gene name** | **Annotation** | **Ratio (POF vs WT)** | **Ratio (5TP vs POF)** |
| --- | --- | --- | --- | --- |
| ENSMUSG00000091736 | Yy2 | transcription factor YY2 [Mus musculus] | 0.04030928 | 36.20069917 |
| ENSMUSG00000046470 | Sox18 | transcription factor SOX-18 [Mus pahari] | 0.289369721 | 3.301647705 |
| ENSMUSG00000040270 | Bach2 | transcription regulator protein BACH2 [Mus musculus] | 0.266282976 | 3.300984135 |
| ENSMUSG00000031229 | Atrx | transcriptional regulator ATRX [Mus musculus] | 0.442210645 | 2.628564822 |
| ENSMUSG00000040929 | Rfx3 | PREDICTED: transcription factor RFX3 isoform X3 [Mus musculus] | 0.332377174 | 2.550834843 |
| ENSMUSG00000049658 | Bdp1 | PREDICTED: transcription factor TFIIIB component B&apos;&apos; homolog isoform X1 [Mus musculus] | 0.344971542 | 2.517339289 |
| ENSMUSG00000024985 | Tcf7l2 | transcription factor 7-like 2 isoform 13 [Mus musculus] | 0.393998467 | 2.505984543 |
| ENSMUSG00000055320 | Tead1 | PREDICTED: transcriptional enhancer factor TEF-1 isoform X1 [Rattus norvegicus] | 0.28808155 | 2.463152246 |
| ENSMUSG00000040732 | Erg | transcriptional regulator ERG isoform 1 [Mus musculus] | 0.212414093 | 2.37822264 |
| ENSMUSG00000053552 | Ebf4 | PREDICTED: transcription factor COE4 isoform X1 [Mus musculus] | 0.912073338 | 2.273597636 |
| ENSMUSG00000048385 | Scrt1 | transcriptional repressor scratch 1 isoform X1 [Meriones unguiculatus] | 1.007275733 | 2.207769866 |
| ENSMUSG00000021359 | Tfap2a | transcription factor AP-2-alpha isoform X2 [Mus caroli] | 0.25210821 | 2.202839346 |
| ENSMUSG00000020962 | Gtf2a1 | transcription initiation factor IIA subunit 1 isoform 1 [Mus musculus] | 0.442422686 | 2.175254654 |
| ENSMUSG00000030199 | Etv6 | transcription factor ETV6 isoform 1 [Mus musculus] | 0.41381827 | 2.156894487 |
| ENSMUSG00000040489 | Sox30 | transcription factor SOX-30 [Mus caroli] | 0.778004581 | 2.14718606 |
| ENSMUSG00000043991 | Pura | PREDICTED: transcriptional activator protein Pur-alpha, partial [Cricetulus griseus] | 0.44423022 | 2.131964256 |
| ENSMUSG00000021318 | Gli3 | transcriptional activator GLI3 [Mus musculus] | 0.409125432 | 2.11904182 |
| ENSMUSG00000041540 | Sox5 | transcription factor SOX-5 isoform a [Mus musculus] | 0.736142766 | 2.081523842 |
| ENSMUSG00000010476 | Ebf3 | transcription factor COE3 [Rattus norvegicus] | 0.371342318 | 2.034215979 |
| ENSMUSG00000042390 | Gatad2b | transcriptional repressor p66-beta [Mus musculus] | 0.556332593 | 2.029401431 |
| ENSMUSG00000001280 | Sp1 | transcription factor Sp1 [Mus musculus] | 0.499911271 | 2.028229561 |
| ENSMUSG00000028634 | Hivep3 | transcription factor HIVEP3 [Mus musculus] | 0.264994337 | 2.026827615 |
| ENSMUSG00000025323 | Sp4 | trans-acting transcription factor 4, partial [Mus musculus] | 0.398648271 | 2.016717965 |
| ENSMUSG00000094483 | Purb | transcriptional activator protein Pur-beta [Mus caroli] | 0.425905914 | 1.868868842 |
| ENSMUSG00000031314 | Taf1 | PREDICTED: transcription initiation factor TFIID subunit 1 isoform X6 [Mus musculus] | 0.583421525 | 1.836425346 |
| ENSMUSG00000063632 | Sox11 | transcription factor SOX-11 [Mesocricetus auratus] | 0.278987536 | 1.813488962 |
| ENSMUSG00000048047 | Zbtb33 | zinc finger and BTB domain containing 33, isoform CRA_b, partial [Mus musculus] | 0.519596848 | 1.799676615 |
| ENSMUSG00000051510 | Mafg | transcription factor MafG isoform X2 [Cavia porcellus] | 0.551373533 | 1.772861252 |
| ENSMUSG00000025612 | Bach1 | transcription regulator protein BACH1 [Mus musculus] | 0.502460898 | 1.772571433 |
| ENSMUSG00000042477 | Tfap2e | transcription factor AP-2-epsilon [Mus musculus] | 1.157501023 | 1.70453398 |
| ENSMUSG00000049038 | Mterf2 | transcription termination factor 2, mitochondrial precursor [Mus musculus] | 0.878457357 | 1.654874381 |
| ENSMUSG00000037343 | Taf2 | transcription initiation factor TFIID subunit 2 [Mus musculus] | 0.536836231 | 1.643891668 |
| ENSMUSG00000022053 | Ebf2 | transcription factor COE2 [Mus musculus] | 0.620637165 | 1.636665581 |
| ENSMUSG00000053477 | Tcf4 | unnamed protein product [Mus musculus] | 1.179343542 | 1.613240816 |
| ENSMUSG00000027109 | Sp3 | transcription factor Sp3 isoform 1 [Mus musculus] | 0.771219796 | 1.613223162 |
| ENSMUSG00000057098 | Ebf1 | early B-cell factor 1, isoform CRA_c, partial [Mus musculus] | 0.776906913 | 1.599240023 |
| ENSMUSG00000019982 | Myb | transcriptional activator Myb isoform 1 [Mus musculus] | 1.120894573 | 1.590710872 |
| ENSMUSG00000041852 | Tcf20 | transcription factor 20 isoform a [Mus musculus] | 0.707840224 | 1.578600708 |
| ENSMUSG00000042507 | Elmsan1 | ELM2 and SANT domain-containing protein 1 [Mus musculus] | 0.492447078 | 1.559709977 |
| ENSMUSG00000020185 | E2f7 | transcription factor E2F7 [Mus musculus] | 0.432654065 | 1.558426015 |
| ENSMUSG00000063060 | Sox7 | transcription factor SOX-7 [Mus musculus] | 7.783436963 | 1.548771989 |
| ENSMUSG00000076431 | Sox4 | transcription factor SOX-4 [Mus pahari] | 0.753524184 | 1.517624254 |
| ENSMUSG00000026803 | Ttf1 | transcription termination factor 1 [Mus musculus] | 0.671219045 | 1.514144489 |
| ENSMUSG00000033006 | Sox10 | LOW QUALITY PROTEIN: transcription factor SOX-10 [Mus caroli] | 0.153712963 | 1.507304752 |
| ENSMUSG00000091243 | Vgll3 | transcription cofactor vestigial-like protein 3 [Mus musculus] | 0.532555222 | 1.501787737 |
| ENSMUSG00000026380 | Tfcp2l1 | transcription factor CP2-like protein 1 [Mus musculus] | 0.277991743 | 1.480465503 |
| ENSMUSG00000070576 | Mn1 | transcriptional activator MN1 [Mus musculus] | 1.196489383 | 1.471570163 |
| ENSMUSG00000038560 | Sp6 | transcription factor Sp6 [Mus musculus] | 0.587802336 | 1.465131468 |
| ENSMUSG00000015501 | Hivep2 | transcription factor HIVEP2 [Mus musculus] | 0.690060363 | 1.464399413 |
| ENSMUSG00000055799 | Tcf7l1 | transcription factor 7-like 1 isoform 1 [Mus musculus] | 0.720787846 | 1.461558118 |
| ENSMUSG00000032411 | Tfdp2 | PREDICTED: transcription factor Dp-2 isoform X2 [Mus musculus] | 0.736825976 | 1.444736752 |
| ENSMUSG00000059540 | Tcea2 | transcription elongation factor A protein 2 isoform a [Mus musculus] | 1.105343772 | 1.438568175 |
| ENSMUSG00000016477 | E2f3 | transcription factor E2F3 isoform E2f3a [Mus musculus] | 0.54630791 | 1.431264407 |
| ENSMUSG00000006642 | Tcf23 | transcription factor 23 [Mus musculus] | 0.485914436 | 1.422584109 |
| ENSMUSG00000000567 | Sox9 | mKIAA4243 protein, partial [Mus musculus] | 0.381710404 | 1.410454163 |
| ENSMUSG00000039117 | Taf4a | transcription initiation factor TFIID subunit 4 [Mus musculus] | 0.471132805 | 1.410295688 |
| ENSMUSG00000024498 | Tcerg1 | PREDICTED: transcription elongation regulator 1 isoform X2 [Microtus ochrogaster] | 0.695663221 | 1.392018753 |
| ENSMUSG00000072889 | Nfxl1 | NF-X1-type zinc finger protein NFXL1 isoform 1 [Mus musculus] | 0.621221409 | 1.380254256 |
| ENSMUSG00000070643 | Sox13 | PREDICTED: transcription factor SOX-13 isoform X2 [Mus musculus] | 0.743646473 | 1.371431856 |
| ENSMUSG00000054321 | Taf4b | transcription initiation factor TFIID subunit 4B [Mus musculus] | 0.439871619 | 1.370875638 |
| ENSMUSG00000027751 | Supt20 | PREDICTED: transcription factor SPT20 homolog isoform X6 [Mus musculus] | 0.963196387 | 1.360156702 |
| ENSMUSG00000053110 | Yap1 | transcriptional coactivator YAP1 isoform 1 [Mus musculus] | 0.729206943 | 1.351005201 |
| ENSMUSG00000074622 | Mafb | transcription factor MafB [Mus musculus] | 0.41938923 | 1.343810128 |
| ENSMUSG00000055435 | Maf | transcription factor Maf [Mus pahari] | 0.541415029 | 1.340106744 |
| ENSMUSG00000029196 | Tada2b | transcriptional adapter 2-beta [Rattus norvegicus] | 0.536488876 | 1.33401186 |
| ENSMUSG00000032228 | Tcf12 | transcription factor 12 isoform 1 [Mus musculus] | 0.874124831 | 1.319930792 |
| ENSMUSG00000099032 | Tcf24 | transcription factor 24 [Mus caroli] | 0.856487893 | 1.319443653 |
| ENSMUSG00000005698 | Ctcf | PREDICTED: transcriptional repressor CTCF isoform X1 [Mus musculus] | 0.642251721 | 1.297993273 |
| ENSMUSG00000028423 | Nfx1 | transcriptional repressor NF-X1 isoform 1 [Mus musculus] | 0.731990187 | 1.276690394 |
| ENSMUSG00000029833 | Trim24 | transcription intermediary factor 1-alpha isoform 1 [Mus musculus] | 0.708585877 | 1.270125773 |
| ENSMUSG00000002249 | Tead3 | transcriptional enhancer factor TEF-5 isoform 1 [Mus musculus] | 1.163682494 | 1.266856259 |
| ENSMUSG00000034968 | Lbx2 | Ladybird homeobox homolog 2 (Drosophila) [Mus musculus] | 0.830171229 | 1.264452682 |
| ENSMUSG00000046179 | E2f8 | transcription factor E2F8 [Mus musculus] | 0.6592585 | 1.2377405 |
| ENSMUSG00000002052 | Supt6 | transcription elongation factor SPT6 [Mus musculus] | 0.652692411 | 1.236520612 |
| ENSMUSG00000052748 | Swt1 | transcriptional protein SWT1 [Mus musculus] | 0.609677103 | 1.228299562 |
| ENSMUSG00000033813 | Tcea1 | transcription elongation factor A protein 1 isoform 1 [Mus musculus] | 0.791101481 | 1.220719806 |
| ENSMUSG00000009596 | Taf7l | transcription initiation factor TFIID subunit 7-like [Mus musculus] | 0.736494799 | 1.190078461 |
| ENSMUSG00000025782 | Taf3 | unnamed protein product [Mus musculus] | 0.589866954 | 1.188793424 |
| ENSMUSG00000038482 | Tfdp1 | transcription factor Dp-1 isoform a [Mus musculus] | 0.810477568 | 1.1814745 |
| ENSMUSG00000053178 | Mterf1b | transcription termination factor 1b, mitochondrial [Mus musculus] | 0.875549971 | 1.175576364 |
| ENSMUSG00000018651 | Tada2a | transcriptional adapter 2-alpha [Mus musculus] | 0.798264941 | 1.160608603 |
| ENSMUSG00000030353 | Tead4 | TEA domain family member 4, isoform CRA_f, partial [Mus musculus] | 0.568621499 | 1.1455008 |
| ENSMUSG00000030315 | Vgll4 | transcription cofactor vestigial-like protein 4 isoform 1 [Mus musculus] | 0.692709606 | 1.144753633 |
| ENSMUSG00000025049 | Taf5 | transcription initiation factor TFIID subunit 5 [Mus musculus] | 0.8368974 | 1.122166171 |
| ENSMUSG00000036180 | Gatad2a | transcriptional repressor p66 alpha isoform a [Mus musculus] | 0.73570297 | 1.110771056 |
| ENSMUSG00000027490 | E2f1 | transcription factor E2F1 isoform a [Mus musculus] | 0.845800899 | 1.105762691 |
| ENSMUSG00000018678 | Sp2 | transcription factor Sp2 isoform 1 [Mus musculus] | 1.004828731 | 1.10550427 |
| ENSMUSG00000021264 | Yy1 | transcriptional repressor protein YY1 [Mus caroli] | 0.953820652 | 1.098780964 |
| ENSMUSG00000026563 | Tada1 | transcriptional adapter 1 [Mus musculus] | 0.70630687 | 1.09079978 |
| ENSMUSG00000045680 | Tcf21 | transcription factor 21 [Mus musculus] | 1.244800815 | 1.088781984 |
| ENSMUSG00000028568 | Btf3l4 | transcription factor BTF3 homolog 4 [Mus musculus] | 1.095653185 | 1.080986091 |
| ENSMUSG00000047242 | Taf9b | transcription initiation factor TFIID subunit 9B isoform 1 [Mus musculus] | 0.677227667 | 1.072989914 |
| ENSMUSG00000033543 | Gtf2a2 | transcription initiation factor IIA subunit 2 [Rattus norvegicus] | 0.793592241 | 1.06166472 |
| ENSMUSG00000054034 | Tceal5 | transcription elongation factor A protein-like 5 [Mus musculus] | 3.766278347 | 1.060004919 |
| ENSMUSG00000011158 | Brf1 | transcription factor IIIB 90 kDa subunit [Mus musculus] | 0.75601581 | 1.053309895 |
| ENSMUSG00000033222 | Ttf2 | transcription termination factor 2 [Mus musculus] | 0.708542433 | 1.050444866 |
| ENSMUSG00000005836 | Gata6 | transcription factor GATA-6 [Mus musculus] | 0.897964649 | 1.041134297 |
| ENSMUSG00000021519 | Mterf3 | transcription termination factor 3, mitochondrial precursor [Mus musculus] | 0.781903765 | 1.036527145 |
| ENSMUSG00000051224 | Tceanc | transcription elongation factor A N-terminal and central domain-containing protein [Mus musculus] | 1.044755688 | 1.023778618 |
| ENSMUSG00000018983 | E2f2 | transcription factor E2F2 isoform 1 [Mus musculus] | 0.876316192 | 1.010188294 |
| ENSMUSG00000027552 | E2f5 | E2F transcription factor 5, isoform CRA_b [Mus musculus] | 1.070523406 | 1.00588076 |
| ENSMUSG00000046909 | Tefm | transcription elongation factor, mitochondrial [Mus musculus] | 1.064135412 | 1.001320452 |
| ENSMUSG00000051817 | Sox12 | PREDICTED: transcription factor SOX-12, partial [Peromyscus maniculatus bairdii] | 1.294200631 | 1.000765844 |
| ENSMUSG00000023980 | Taf8 | transcription initiation factor TFIID subunit 8 isoform 1 [Mus musculus] | 0.862199334 | 0.99598255 |
| ENSMUSG00000057469 | E2f6 | transcription factor E2F6 [Mus musculus] | 0.827252407 | 0.98279403 |
| ENSMUSG00000048100 | Taf13 | transcription initiation factor TFIID subunit 13 [Heterocephalus glaber] | 1.020472481 | 0.970495785 |
| ENSMUSG00000023781 | Hes7 | transcription factor HES-7 [Mus musculus] | 1.167618991 | 0.968183363 |
| ENSMUSG00000020167 | Tcf3 | transcription factor E2-alpha isoform 1 [Mus musculus] | 1.183523038 | 0.960120506 |
| ENSMUSG00000044550 | Tceal3 | transcription elongation factor A protein-like 3 [Mus musculus] | 3.297328885 | 0.959305264 |
| ENSMUSG00000003923 | Tfam | transcription factor A, mitochondrial precursor [Mus musculus] | 0.828859242 | 0.956432254 |
| ENSMUSG00000040429 | Mterf1a | PREDICTED: transcription termination factor 1a, mitochondrial isoform X1 [Mus musculus] | 1.248926555 | 0.953872144 |
| ENSMUSG00000036980 | Taf6 | TAF6 RNA polymerase II, TATA box binding protein (TBP)-associated factor, isoform CRA_b, partial [Mus musculus] | 0.966664981 | 0.953712 |
| ENSMUSG00000018143 | Mafk | transcription factor MafK [Mus musculus] | 0.506482145 | 0.951692484 |
| ENSMUSG00000074637 | Sox2 | PREDICTED: transcription factor SOX-2 [Microtus ochrogaster] | 0.426140313 | 0.949336512 |
| ENSMUSG00000001604 | Tcea3 | transcription elongation factor A protein 3 [Mus musculus] | 1.592516801 | 0.947744436 |
| ENSMUSG00000049536 | Tceal1 | transcription elongation factor A protein-like 1 [Mus musculus] | 1.37564349 | 0.947554509 |
| ENSMUSG00000003435 | Supt5 | transcription elongation factor SPT5 [Rattus norvegicus] | 0.901833492 | 0.9269738 |
| ENSMUSG00000042712 | Wbp5 | transcription elongation factor A protein-like 9 [Mus musculus] | 1.363818012 | 0.926908672 |
| ENSMUSG00000000782 | Tcf7 | transcription factor 7 [Mus musculus] | 1.376556029 | 0.924739934 |
| ENSMUSG00000022528 | Hes1 | transcription factor HES-1 [Meriones unguiculatus] | 1.637910514 | 0.911776671 |
| ENSMUSG00000052684 | Jun | transcription factor AP-1 [Ictidomys tridecemlineatus] | 0.56684181 | 0.907934521 |
| ENSMUSG00000000134 | Tfe3 | unnamed protein product [Mus musculus] | 0.86080555 | 0.906350738 |
| ENSMUSG00000014859 | E2f4 | transcription factor E2F4 [Mus musculus] | 0.950082957 | 0.89425696 |
| ENSMUSG00000001472 | Tcf25 | unnamed protein product [Mus musculus] | 1.216938207 | 0.887267121 |
| ENSMUSG00000048930 | Tada3 | transcriptional adaptor 3 (NGG1 homolog, yeast)-like, isoform CRA_c, partial [Mus musculus] | 1.248103594 | 0.881747053 |
| ENSMUSG00000024137 | E4f1 | transcription factor E4F1 isoform 2 [Mus musculus] | 1.061221368 | 0.875869278 |
| ENSMUSG00000060284 | Sp7 | transcription factor Sp7 isoform 1 [Mus musculus] | 3.371013725 | 0.875647136 |
| ENSMUSG00000024218 | Taf11 | transcription initiation factor TFIID subunit 11 [Mus musculus] | 1.139028982 | 0.872970921 |
| ENSMUSG00000051316 | Taf7 | transcription initiation factor TFIID subunit 7 [Mus musculus] | 0.734081567 | 0.870609988 |
| ENSMUSG00000058794 | Nfe2 | PREDICTED: transcription factor NF-E2 45 kDa subunit isoform X1 [Mus musculus] | 1.29502016 | 0.869402774 |
| ENSMUSG00000050410 | Tcf19 | transcription factor 19-like protein [Mus musculus] | 0.951924282 | 0.845622734 |
| ENSMUSG00000024927 | Rela | transcription factor p65 [Mus musculus] | 0.987285935 | 0.841865035 |
| ENSMUSG00000051579 | Tceal8 | transcription elongation factor A protein-like 8 [Mus musculus] | 1.493876533 | 0.838982983 |
| ENSMUSG00000028271 | Gtf2b | transcription initiation factor IIB [Rattus norvegicus] | 1.036706295 | 0.818295721 |
| ENSMUSG00000023990 | Tfeb | transcription factor EB isoform a [Mus musculus] | 1.011733182 | 0.817601985 |
| ENSMUSG00000028899 | Taf12 | PREDICTED: transcription initiation factor TFIID subunit 12 isoform X1 [Peromyscus maniculatus bairdii] | 0.864246768 | 0.813593857 |
| ENSMUSG00000005566 | Trim28 | KRAB-A interacting protein [Mus musculus] | 0.980018167 | 0.809840807 |
| ENSMUSG00000042622 | Maff | PREDICTED: transcription factor MafF isoform X1 [Peromyscus maniculatus bairdii] | 1.350360353 | 0.796438902 |
| ENSMUSG00000078941 | Ak6 | transcription initiation factor TFIID subunit 9 [Mus musculus] | 1.163903524 | 0.7944146 |
| ENSMUSG00000004359 | Spic | transcription factor Spi-C [Mus musculus] | 1.705079314 | 0.784710485 |
| ENSMUSG00000071076 | Jund | PREDICTED: transcription factor jun-D [Peromyscus maniculatus bairdii] | 1.375971623 | 0.778468209 |
| ENSMUSG00000028619 | Tceanc2 | transcription elongation factor A N-terminal and central domain-containing protein 2 [Mus musculus] | 1.181949112 | 0.767854977 |
| ENSMUSG00000026273 | Mterf4 | transcription termination factor 4, mitochondrial isoform 1 [Mus musculus] | 1.183368461 | 0.748287867 |
| ENSMUSG00000067071 | Hes6 | transcription cofactor HES-6 isoform 1 [Mus musculus] | 1.287419461 | 0.740239256 |
| ENSMUSG00000031487 | Brf2 | transcription factor IIIB 50 kDa subunit [Mus musculus] | 1.037817402 | 0.739375139 |
| ENSMUSG00000021660 | Btf3 | transcription factor BTF3 isoform 1 [Mus musculus] | 1.389884032 | 0.733737053 |
| ENSMUSG00000021944 | Gata4 | transcription factor GATA-4 [Mus musculus] | 1.70248796 | 0.731981 |
| ENSMUSG00000024176 | Sox8 | transcription factor SOX-8 [Mus pahari] | 0.429731328 | 0.730118161 |
| ENSMUSG00000030796 | Tead2 | unnamed protein product [Mus musculus] | 1.701011875 | 0.721509986 |
| ENSMUSG00000043866 | Taf10 | hypothetical protein A6R68_21217 [Neotoma lepida] | 1.703355384 | 0.698132426 |
| ENSMUSG00000016503 | Gtf3a | general transcription factor III A, isoform CRA_a, partial [Mus musculus] | 1.307750264 | 0.697784917 |
| ENSMUSG00000029553 | Tfec | PREDICTED: transcription factor EC isoform X1 [Mus musculus] | 0.865309784 | 0.694258236 |
| ENSMUSG00000052837 | Junb | transcription factor jun-B [Mus musculus] | 1.111500056 | 0.654206795 |
| ENSMUSG00000002983 | Relb | transcription factor RelB isoform X1 [Mus pahari] | 1.027716559 | 0.643449384 |
| ENSMUSG00000052293 | Taf9 | transcription initiation factor TFIID subunit 9 [Mus musculus] | 1.651425269 | 0.638131882 |
| ENSMUSG00000002111 | Spi1 | transcription factor PU.1 [Mus musculus] | 0.667487182 | 0.637016097 |
| ENSMUSG00000071151 | Gm4799 | transcription initiation factor TFIID subunit 10 [Mus musculus] | 0.595913328 | 0.612094395 |
| ENSMUSG00000020485 | Supt4a | transcription elongation factor SPT4 [Mus caroli] | 1.031619318 | 0.604073125 |
| ENSMUSG00000013822 | Elof1 | EGF-like module-containing mucin-like hormone receptor-like 4 [Fukomys damarensis] | 1.220271885 | 0.586246007 |
| ENSMUSG00000047591 | Mafa | transcription factor MafA [Mus musculus] | 1.017582301 | 0.561753642 |
| ENSMUSG00000025902 | Sox17 | SRY-box containing gene 17, isoform CRA_b, partial [Mus musculus] | 0.739792678 | 0 |
| ENSMUSG00000051910 | Sox6 | transcription factor SOX-6 isoform 1 [Mus musculus] | 0.269813971 | 0 |
