# Supplementary material for: Thymopentin alleviates premature ovarian failure in mice by activating YY2/Lin28A and inhibiting the expression of let‐7 family microRNAs
Source: Cell Prolif. 2021 Jun 28;54(8):e13089. doi: 10.1111/cpr.13089 (PMC8349654; doi:10.1111/cpr.13089)
Supplement: Supplementary file 5 — Supplementary Material [file CPR-54-e13089-s001.docx]

**Thymopentin alleviates premature ovarian failure in mice by activating YY2/Lin28A and inhibiting the expression of let-7 family microRNAs**

**2. Materials and methods**

A detailed description of all materials and methods can be found in Supplementary Materials and Methods.

***2.1 HFHS-POF model and TP-5 treatment***

This model derives from previously published methods ^1-4^. Briefly, 10-week-old female C57BL/6 mice (n = 30) were purchased from the Experimental Animal Centre of the Shanghai University of Traditional Chinese Medicine. Mice were randomly allocated to three groups, with ten mice per group. WT mice in the blank control group were fed with a normal diet without any intervention, while the mice in the treatment group (POF-5TP) were fed with a high-fat diet (8 g/kg) and received 200 μl of 30% fructose and an intraperitoneal injection of 5 mg/kg TP-5 once per day (Hainan Zhonghe Pharmaceutical Co., Ltd., Hainan, China). The mice in the model group (POF-Saline) were fed with a high-fat diet (8 g/kg) and received 200 μl of 30% fructose and an intraperitoneal injection of normal saline once per day. Mice in each group were administered treatment continuously for 30 days. Our protocol was approved by the Ethics Committee of the Shanghai Institute of Traditional Chinese Medicine and Geriatrics (no. SHAGESYDW202009). All of the experiments complied with the regulations on Experimental Animals of the State Science and Technology Commission of China.

***2.2 SiRNA loading of PLGA nanoparticles and interventions in animal models***

Our methods were performed according to previous reports ^4-7^. Briefly, YY2-siRNA (siYY2) and random control (siMock) oligoRNAs were synthesized by Genepharma (Genepharma, Shanghai, China). The PLGA (MedChemExpress, Shanghai, China) was dissolved overnight in methylene chloride, prior to siYY2/ siMOCK and spermidine complex formation using an 8:1 molar ratio of the polyamine nitrogen to nucleotide phosphate. One hundred nanomoles of siYY2 or siMOCK per 100 mg of polymer in Tris-EDTA (10 mM Tris-HCl and 1 mM EDTA) buffer (Sigma-Aldrich, St. Louis, USA) were added dropwise to the PLGA solution while vortexing. This solution was sonicated and subsequently added to 2.5% polyvinyl alcohol and a 5 mg/mL avidin-palmitate solution for the second emulsion. The nanoparticles were hardened during solvent evaporation in 0.3% polyvinyl alcohol for 3 h. To synthesize unmodified nanoparticles, the second emulsion contained only 2.5% polyvinyl alcohol, and nanoparticles were incubated post-hardening in PBS without ligand for 30 min. All of the nanoparticles were washed twice in deionized water to remove residual solvent, centrifuged at 4°C, lyophilized, and stored at −20°C. As previously reported ^4-7^, 5 mg of siRNA@PLGA was dissolved in 0.5 mL of methylene chloride for 30 min, and siYY2/siMOCK was extracted twice into Tris–EDTA buffer. Encapsulation efficiency was determined by comparing the amount of siRNA loaded onto the PLGA nanoparticles with a theoretical loading of 1 nmol siRNA/mg polymer. For the siRNA@PLGA (nanoparticle-siRNA-CH2.5), the loading was 514 pmol siRNA/mg nanoparticle. The administered dosage of siRNA@PLGA was 400 μl (20 mg/ml) once every 2 days.

***2.3 RNA extraction, reverse transcription, and qPCR detection***

Total RNA was extracted according to the Trizol reagent instructions (Invitrogen). Total RNA from each group was extracted and treated with DNase I (Sigma-Aldrich), and then quantified and reversed transcribed with ReverTra Ace-α first-strand and cDNA Synthesis Kit (TOYOBO) to produce cDNA. QRT-PCR was completed using a Realplex4 Real-Time PCR detection system (Eppendorf Co., Ltd., Germany), and a SYBR Green Real-Time PCR Master Mix (Toyobo) was used as the fluorescent dye for nucleic acid amplification. We employed a total of 40 QRT-PCR amplification cycles: denaturation at 95°C, 15 s at 58°C, annealing at 58°C for 30 s, and primer template extension at 72°C for 42 s. We determined relative gene expression using the 2-delta Ct calculation method, in which the expression level of ΔCt = Ct_genes–Ct_18sRNA; ΔCt = ΔCt_all_groups–ΔCt_blank control_group. The mRNA was corrected according to the expression level of 18s rRNA. The primers needed for each gene amplification are depicted in Table S1.

***2.4 Western immunoblotting analysis***

Briefly, the total proteins from each group of cells were electrophoresed using 12% SDS-PAGE denaturing gel, and then transferred to a PVDF membrane (Millipore). After sealing and washing, the membrane was incubated with primary antibodies (Table S2) at 37°C and allowed to react for 45 min. After thoroughly washing, the membrane was incubated with second antibodies for a reaction time of 45 min (Table S2) at 37°C. We washed the membrane with TBST four times at room temperature for 14 min each time. We then used enhanced chemiluminescence (ECL Kit, Pierce Biotechnology) and exposed and developed the film (Sigma-Aldrich Chemical).

***2.5 Isolation and culture of mouse ovarian granulosa cells***

In accordance with our previous studies ^4,8-10^, 10-week-old female C57BL/6 mice (n = 10) were purchased from the Experimental Animal Centre of the Shanghai University of Traditional Chinese Medicine, and sacrificed by cervical dislocation. Ovarian tissues were isolated under sterile conditions and were placed in phosphate-buffered saline (PBS) at 4°C. The ovarian tissues were minced, and 2.0 ml of hyaluronidase (0.1%, Sigma-Aldrich, St Louis, MO, USA) was added to the tissues for 1 min of digestion at 37°C. The tissue suspension was gently pipetted, and 200 μl of fetal calf serum (Gibco, Gaithersburg, MD, USA) was added to the suspension to terminate the digestion; the suspension was then filtered through a 200-mesh cell strainer. Next, 5.0 ml of PBS was added to the filtrate and mixed well, followed by centrifugation at 1500 r/min for 5 min at 10°C. The supernatant was discarded, and the pellet was re-suspended in 5.0 ml of PBS, followed by centrifugation at 1500 r/min for 5 min at 10°C. The supernatant was again discarded, and the cell pellet was re-suspended in Dulbecco's Modified Eagle’s Medium: Ham’s F-12 medium (DMEM:F12) (1:1) and mixed well. The medium contained 10% fetal bovine serum, 10 ng/ml basic fibroblast growth factor (bFGF), 10 ng/ml epidermal growth factor (EGF), 2 mM L-glutamine, 10 ng/ml growth hormone (GH), and 15 ng/ml estradiol (E2) (Gibco, Gaithersburg, MD, USA). The cellular suspension was seeded in 6-well cell culture plates, which was then incubated at 37°C in 5% CO_2_ in compressed air at high humidity until the cells achieved 80% confluency.

***2.6 Luciferase reporter assay***

Luciferase reporter assays were performed as previously described ^11^. The mOGCs were seeded at 30,000/well in 48-well plates and co-transfected with 20 ng of psiCHECK-WT-YY2-P (insert fragment, 5'-CACGGGCTCAGCAGACGA**CCATGG**GCTCGGTGTCCAACCAGCAG-3'); psiCHECK-Mut-YY2-P (insert fragment, 5'-CACGGGCTCAGCAGACGA**CgAcAt**GCTCGGTGTCCAACCAGCAG-3') or psiCHECK-Blank (Novobiosci, Shanghai, China) using Lipofectamine 2000 according to the manufacturer’s protocol. Luciferase activity was measured after 48 h using the Dual-Luciferase Reporter Assay System (Promega, Madison, USA).

***2.7 Hematoxylin-eosin staining***

In brief, all of the fresh tissues were immersed in 4% paraformaldehyde (Sigma Aldrich, St. Louis, USA) at room temperature for 30 min, dehydrated through a graded series of ethanol, embedded in paraffin, sectioned at 6 μm, and slides soaked in xylene for dewaxing. Histologic sections were stained with hematoxylin-eosin (H&E, Sigma Aldrich, St. Louis, USA) and finally coated with xylene (Sigma Aldrich, St. Louis, USA) and neutral resin (Sigma Aldrich, St. Louis, USA).

***2.8 HPLC-MS/MS assay***

A 250-μl sample (calibration working solution or plasma) was mixed with 25 μl of IS working solution and 250 μl of methanol, and we rotated the mixture for 2 min. We then added 250 μl of deionized water to the mixture and shook the mixture for 1 min. After 10,000 g centrifugation for 5 min, the mixture was transferred to the HLB elution plate, and 600 μl of extraction supernatant was added. The supernatant slowly flowed through the SPE board under negative pressure and entered the waste drum. Acetonitrile/ddH2O and 200 μl of n-hexane were added to the solid-phase extraction plate sequentially. The waste container was replaced by a 96-hole plate, and 30 μl of methanol/acetonitrile (1/9/v/v) was added to the solid-phase extraction plate to collect the filtrate. The filtrate was diluted with 50 μl of ddH2O and agitated. The filtrate was then diluted in a 96-well plate and immediately analyzed by liquid chromatography-mass spectrometry/mass spectrometry. The 10-ul filtrate was injected into the water at Teyote Tex, and the temperature of the LC-MS analysis system and the automatic sampler was 10°C. The binary mobile phase consisted of 0.3-mm NH_4_F (A) and methanol (B). With the column temperature maintained at 35°C, the optimal mass spectrometric parameters were a capillary voltage of 3.56 kV, cone voltage of 50 V, source temperature of 150°C, desolvation temperature of 600 C, cone gas flow of 150 L/h, desolvation gas flow of 1000 L/h, and collision gas flow of 0.15 ml/min. Steroids were analyzed by positive- and negative-ion modes, combined with electrospray ionization mass spectrometry (ESI-MS) and tandem mass spectrometry (MS/MS). We quantified 15 analytes using multi-reaction monitoring (MRM), and 5 analytes (E1, E2, E3, ALDO, and 17-OH-PR) using the negative-ion mode.

***2.9 Immunofluorescence staining***

In brief, all of the fresh tissues were soaked at room temperature and fixed in 4% paraformaldehyde (Sigma-Aldrich, St. Louis, USA) for 30 min. We performed ethanol-gradient dehydration, paraffin embedding, tissue sectioning at a thickness of 6 μm), and dewaxing in xylene. The tissue sections were sealed at 37°C for 30 min with immunohistochemical blocking solution (Beyotime Biotechnology Co., Ltd., Zhejiang, China). We discarded the blocking solution and added immunohistochemical cleaning solution (Beyotime Biotechnology Co., Ltd., Zhejiang, China) to rinse sections three times at room temperature for 5 min each. Primary antibodies (Table S2) were added and incubated at 37°C for 45 min. We discarded the antibodies and added immunohistochemical cleaning solution (Beyotime Biotechnology Co., Ltd., Zhejiang, China) to rinse three times at room temperature for 5 min each. Then, the secondary antibodies (Table S2) were added and we incubated sections at 37°C for 45 min. After discarding the antibody, we added the immunized histochemical cleaning solution (Beyotime Biotechnology Co., Ltd., Zhejiang, China) and rinsed at room temperature for 5 min 3 times. Finally, an immunofluorescence sealing solution (Sigma-Aldrich, St. Louis, USA) was added to seal the tablets.

***2.10 Establishment of cDNA sequencing libraries and high-throughput RNA-Seq***

The following analysis was executed by KangChen Biotech (Shanghai, China). In accordance with their experimental procedures, a random-fragment sequencing library was constructed using a SOLiD Whole Transcriptome Analysis Kit (Life Technologies). Nucleic acid cleaving reagents were added, and the mRNA was randomly disrupted into short segments in a shaking incubator. First-strand cDNA was reverse transcribed using the fragmented mRNA as the template; and second-strand cDNA was synthesized using a second-strand DNA-synthesis reaction system consisting of DNA polymerase I, dNTPs, and RNase H (Sigma). The synthesized DNA was purified using a DNA purification kit and recovered. The base “A” was added to the 3’end of the cDNA, followed by ligation to the adapter in order to complete the blunt-end repair reaction. Subsequently, we performed DNA fragment-size selection. Finally, the cDNA was used for PCR amplification to obtain a sequencing library. The constructed library was qualified using an Agilent 2100 Bioanalyzer and the ABI StepOnePlus Real-Time PCR System, and it was subjected to high-throughput sequencing using an Illumina HiSeq ™ 2000 Sequencer after passing quality controls.

***2.11 Statistical analysis***

Each experiment was performed at least three times, and data are presented as the mean +/- standard error, where applicable. Differences were evaluated with the Student’s *t*-test. A P value less than 0.05 was considered to be statistically significant.

**References:**

1. Goncalves MD, Lu C, Tutnauer J, et al. High-fructose corn syrup enhances intestinal tumor growth in mice. *Science.* 2019;363(6433):1345-1349.

2. Swider E, Maharjan S, Houkes K, et al. Forster Resonance Energy Transfer-Based Stability Assessment of PLGA Nanoparticles in Vitro and in Vivo. *ACS Appl Bio Mater.* 2019;2(3):1131-1140.

3. Cheng J, Teply BA, Sherifi I, et al. Formulation of functionalized PLGA-PEG nanoparticles for in vivo targeted drug delivery. *Biomaterials.* 2007;28(5):869-876.

4. Liu T, Lin J, Chen C, et al. MicroRNA-146b-5p overexpression attenuates premature ovarian failure in mice by inhibiting the Dab2ip/Ask1/p38-Mapk pathway and gammaH2A.X phosphorylation. *Cell Prolif.* 2021;54(1):e12954.

5. Martin DT, Shen H, Steinbach-Rankins JM, et al. Glycoprotein-130 Expression Is Associated with Aggressive Bladder Cancer and Is a Potential Therapeutic Target. *Mol Cancer Ther.* 2019;18(2):413-420.

6. Martin DT, Steinbach JM, Liu J, et al. Surface-modified nanoparticles enhance transurothelial penetration and delivery of survivin siRNA in treating bladder cancer. *Mol Cancer Ther.* 2014;13(1):71-81.

7. Woodrow KA, Cu Y, Booth CJ, Saucier-Sawyer JK, Wood MJ, Saltzman WM. Intravaginal gene silencing using biodegradable polymer nanoparticles densely loaded with small-interfering RNA. *Nat Mater.* 2009;8(6):526-533.

8. Xiong Y, Liu T, Wang S, Chi H, Chen C, Zheng J. Cyclophosphamide promotes the proliferation inhibition of mouse ovarian granulosa cells and premature ovarian failure by activating the lncRNA-Meg3-p53-p66Shc pathway. *Gene.* 2017;596:1-8.

9. Liu T, Liu Y, Huang Y, et al. miR-15b induces premature ovarian failure in mice via inhibition of alpha-Klotho expression in ovarian granulosa cells. *Free Radic Biol Med.* 2019;141:383-392.

10. Liu T, Huang Y, Zhang J, et al. Transplantation of human menstrual blood stem cells to treat premature ovarian failure in mouse model. *Stem Cells Dev.* 2014;23(13):1548-1557.

11. Cheng W, Liu T, Wan X, Gao Y, Wang H. MicroRNA-199a targets CD44 to suppress the tumorigenicity and multidrug resistance of ovarian cancer-initiating cells. *FEBS J.* 2012;279(11):2047-2059.
